# Clinical Value and Potential Mechanism of miRNA-33a-5p in Lung Squamous Cell Carcinoma

**DOI:** 10.1155/2021/6614331

**Published:** 2021-11-29

**Authors:** Xiang-Ming Wang, Shang-Wei Chen, Gang Chen, Hua-Fu Zhou, Ting-Qing Gan, Jing-Jing Zeng, Zu-Yun Li

**Affiliations:** ^1^Department of Pathology, The First Affiliated Hospital of Guangxi Medical University, Nanning, Guangxi 530021, China; ^2^Department of Cardio-Thoracic Surgery, The First Affiliated Hospital of Guangxi Medical University, Nanning, Guangxi 530021, China; ^3^Department of Medical Oncology, The Second Affiliated Hospital of Guangxi Medical University, Nanning, Guangxi 530021, China

## Abstract

This study is aimed at thoroughly exploring the expression status, clinical significance, and underlying molecular mechanism of miRNA-33a-5p in lung squamous cell carcinoma (LUSC). Here, we detected miRNA-33a-5p in 20 samples from patients with LUSCs and 20 matching non-LUSC specimens by in-house quantitative real-time PCR (RT-qPCR). Relationship between miRNA-33a-5p expression and clinicopathological traits was investigated from materials derived from miRNA sequencing and miRNA microarrays. A pool standard mean difference (SMD) and summary receiver operating characteristic curves (SROC) were calculated to evaluate the integrated expression value of miRNA-33a-5p in LUSC. Twelve online platforms were applied to select potential target genes of miRNA-33a-5p. The differentially expressed genes (DEGs) of LUSC and the candidate target genes of miRNA-33a-5p were overlapped to acquire a set of specific genes for further analyses of the Kyoto Encyclopedia of Genes and Genomes (KEGG), Gene Ontology (GO), and protein–protein interaction (PPI) network. miRNA-33a-5p overexpressed in LUSC was supported by 706 LUSC and 261 non-LUSC samples gathering from RT-qPCR, miRNA-seq, and public miRNA microarrays. The pooled SMD was 0.56 (95% CI: -0.01-1.05), and the area under the curve (AUC) of the SROC was 0.78 (95% CI: 0.74-0.82). A total of 240 genes were identified as potential target genes of miRNA-33a-5p for functional enrichment analyses; the results suggested that these target genes may participate in several vital biological processes that promote the proliferation and progression of LUSC. miRNA-33a-5p may play an essential role in the occurrence and development of LUSC by targeting hub genes (ETS1, EDNRB, CYR61, and LRRK2) derived from the PPI network. In summary, our results indicated that miRNA-33a-5p may contribute as a prospective therapeutic target in LUSC.

## 1. Introduction

Today, regardless of the morbidity or the number of fatalities, lung cancer (LC) is ranked highest among all known cancers in the world. According to the American Cancer Society, it is estimated that there were 228,150 new diagnostic LC cases and 142,670 LC-associated deaths in the United States in 2019, which accounted for 18.4% of all cancer-related deaths in the population [1]. Currently, in comparison to previous traditional therapeutic strategies, including chemotherapy, targeted molecular therapy, immunotherapy, and antivascular therapy, have been very effective in the treatment of LC. Nonetheless, the overall 5% survival rate for advanced LC patients remains dismal [2]. Small-cell lung cancer (SCLC) and non-SCLC (NSCLC) are the two most frequent pathological subtypes of LC. NSCLC accounts for nearly 80–85% in all of patients diagnosed with LC [3]. Pathologically, NSCLC mainly consists of lung adenocarcinoma (LUAD), which is derived from the glandular epithelium of the lung, and lung squamous cell carcinoma (LUSC), which originates from the carcinogenesis of squamous epithelium that has been transformed from the glandular epithelium on the lung tissue. Patients with LUSC have a lower overall survival rate than patients with LUAD, mainly due to the low rate of early discovery and the lack of practical therapeutic solutions [4]. Hence, identification of a promising biomarker is crucial for the diagnosis, treatment, and prognosis prediction for patients with LUSC.

MicroRNA, also known as miRNA, is a type of small noncoding RNA that has a negative regulatory effect on coded proteins. miRNAs are also capable of suppressing the expression of mRNAs and simultaneously destroying them [5]. Through regulation of the oncogenes, miRNAs can act as tumor-inhibiting factors or genes; thus, recently, they have become a novel subject for exploring the molecular mechanisms of multiple cancers. miRNA-33a-5p is an intron miRNA that is located inside the intron sequence of the sterol-response-element-binding protein gene 2 (SREBP2) [6]. A previous study has reported that miRNA-33a-5p had the ability to restrain some of the biological behavior of NSCLC cell lines, such as proliferation and motility [7]. Other previous researches have also illustrated that miRNA-33a-5p was able to affect the molecular mechanisms of esophageal squamous cell carcinoma, hepatocellular carcinoma, colorectal carcinoma, and tongue squamous cell carcinoma [8–11]. However, to date, the detailed molecular mechanism and clinical implication of miRNA-33a-5p with LUSC remain elusive.

In the present study, we applied in-house reverse transcription quantitative polymerase chain reaction (RT-qPCR), TCGA miRNA sequencing (miRNA-seq) data, and miRNA chips of online database to thoroughly investigate the expression status, clinical significance, and underlying molecular mechanism of miRNA-33a-5p in LUSC. Moreover, 12 microRNA online platforms were collectively utilized to preliminarily forecast the potential mRNAs sponged by miRNA-33a-5p. Information from the Gene Ontology (GO) project, the Kyoto Encyclopedia Genes and Genomes (KEGG) database, and protein-protein interactions (PPI) were successively employed to track some of the latent functional mechanisms of miRNA-33a-5p when regulating LUSC tumorigenesis and evolvement.

## 2. Materials and Methods

### 2.1. Clinical Specimens

Before the RT-qPCR experiment was conducted, patients diagnosed with LUSC at the First Affiliated Hospital of Guangxi Medical University from October 2018 to September 2019 were enrolled in this study. These patients received no medication or other treatment before. The samples obtained from patients who have undergone LC radical resection were made into paraffin blocks, and only those with a tumor cell ratio ≥ 75% were involved in the present study. Finally, 20 formalin-fixed, paraffin-embedded (FFPE) LUSC, and matching non-LUSC tissues were acquired from the Department of Pathology. All the LUSC specimens were independently validated by two different pathologists (Zu-Yun Li and Gang Chen). All the study participants signed the sampling informed consent form. The study complied with the Declaration of Helsinki and was authorized by the Ethics Committee of the First Affiliated Hospital of Guangxi Medical University.

### 2.2. In-House RT-qPCR

To examine the expression profile of miRNA-33a-5p in LUSC, extraction, isolation, and normalization total RNA and the RT-qPCR assay were performed as previously reported procedure [12–16]. The specific primer of miRNA-33a-5p was provided by TaqMan microRNA Assays (4427975-000468; Applied Biosystems, Life Technologies Europe B. V, Bleiswijk, Netherlands). The reverse primers were applied in the reverse transcription step with TaqMan microRNA Reverse Transcription Kit (4366596; Applied Biosystems, Life Technologies Europe B.V.). RNU6B was the endogenous control, which acted as the reference gene in previous reports [16–18]. The base sequences for the preceding miRNAs were as follows: miRNA-33a-5p—CTGTGGTGCATTGTAGTTGCATTGCATGTTCTGGTGGTACCCATGCAATGTTTCCACAGTGCATCACAG; RNU6B—CGCAAGGAUGACACGCAAAUUCGUGAAGCGUUCCAUAUUUUU. The expression values of miRNA-33a-5p were uniformly computed according to the 2^-*Δ*Cq^ formula, in which the “Cq” value refers to the quantification cycle number [19].

### 2.3. Extraction of miRNA-33a-5p Expression in LUSC from the Public Database

The relevant data of miRNA-seq and miRNA microarrays were acquired from the Gene Expression Omnibus (GEO) (https://www.ncbi.nim.nih.gov/geo/), Oncomine (https://www.oncomine.org/), ArrayExpress (https://www.ebi.ac.uk/arrayexpress/), SRA (https://www.sra.org.uk/), and The Cancer Genome Atlas (TCGA) (https://tcga-data.nci.nih.gov/docs/publications/tcga/) databases as previously described [12–16, 20]. Then, we extracted the expression value of miRNA-33a-5p from the data which we got from the above-mentioned public online databases. Subsequently, the miRNA-33a-5p expression level in LUSC samples and related clinical features (such as age, gender, and pathological stage) were explored.

### 2.4. Integrated Analysis of miRNA-33a-5p Expression in LUSC

The miRNA-33a-5p expression data on LUSC, derived from in-house RT-qPCR, TCGA, and online microarray datasets, were collectively combined for the comprehensively integrated analysis of the miRNA-33a-5p expression profile in LUSC by R v3.6.1 software. The pooled standardized mean difference (SMD) was calculated to assess the miRNA-33a-5p expression value in LUSC. The pooled heterogeneity among the included datasets was evaluated by *I*-squared (*I*^2^), with its specific *P* value, to decide which pooling model would be used when the SMD value was computed. For example, if the heterogeneity was high (*I*^2^ ≥ 50%; *P* ≤ 0.05), a random effects model was applied; otherwise, a fixed effects model was employed. To thoroughly examine the efficiency of miRNA-33a-5p when distinguishing the LUSC samples from the nontumor samples, the summarized receiver operating characteristic (SROC) curve was delineated.

### 2.5. Prognostic Value of miRNA-33a-5p in LUSC

In order to evaluate the prognosis of miRNA-33a-5p in LUSC patients, the Kaplan–Meier survival curve was established by treating the median expression value of miRNA-33a-5p as a cutoff value. Additionally, the Cox univariate and multivariate regression analyses were carried out to examine whether miRNA-33a-5p could be acted as an independent prognostic factor in LUSC by R v3.6.1 software.

### 2.6. Forecast for the Potential Target mRNAs of miRNA-33a-5p

The target mRNAs of miRNA-33a-5p were forecasted in virtue of 12 miRNA databases in silico as our previous reported study [12–14, 16]. The mRNAs that emerged in more than four miRNA platforms were further selected as potential target mRNAs of miRNA-33a-5p. As the sponge role between miRNA and mRNA, an overexpression of miRNA may lead to a low expression of the target mRNA, so we screened the downexpressed differentially expressed genes (DEGs) from RNA-seq and RNA chips as the candidate target genes of miRNA-33a-5p in LUSC. We filtered all downregulated DEGs in LUSC with the screening threshold were log2 fold change (FC) < −1 and adj. *P* value < 0.05 using “limma” package of R v3.6.1. Then, the predicted target mRNAs and the downexpressed DEGs of LUSC were intersected to acquire the final target mRNAs of miRNA-33a-5p, the latter of which were subsequently utilized to further perform the functional enrichment analysis and identify the hub genes.

### 2.7. Functional Enrichment Analysis for Promising Target mRNAs

Functional enrichment analyses were conducted via the “ClusterProfiler” R package based on the preceding target mRNAs of miRNA-33a-5p. The functional terms with *P* value < 0.05 were considered statistically significant. The PPI network was constructed using the Search Tool for the Retrieval of Interacting Genes (STRING) v. 11.0 platform [21]. Afterwards, a histogram of key genes reflecting the occurrences of the proteins in PPI network was plotted using R language. The relationship between miRNA-33a-5p and the core target mRNAs was also analyzed using R v3.6.1.

### 2.8. Statistical Analysis

All data of the miRNA-seq expression values were transformed to log2(*X* + 1). miRNA-33a-5p expression was exhibited in the form of mean (*M*) ± standard deviation (SD) via SPSS v25.0. Differential expression of miRNA-33a-5p between LUSC and non-LUSC tissues from RT-qPCR and miRNA-seq data was evaluated by paired/unpaired Student's *t*-test or the Mann–Whitney test. When the clinicopathological parameters contained three or more subgroups, the Kruskal–Wallis test was performed to examine the significance of miRNA-33a-5p differential expression. GraphPad Prism v8.0 was applied to draw scatter diagrams and ROC curves. The SROC curve was created by Stata v12.0. The pooled SMD value was computed by R v3.6.1. *P* values less than 0.05 were regarded to be statistically significant.

## 3. Results

### 3.1. miRNA-33a-5p Expression in LUSC according to RT-qPCR

**Figure 1:**
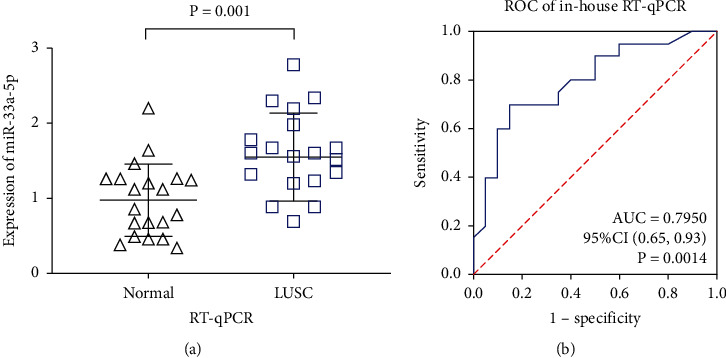
miRNA-33a-5p overexpression in LUSC based on RT-qPCR. (a) The expression level of miRNA-33a-5p in 20 LUSC and 20 normal lung tissues based on in-house RT-qPCR. (b) The ROC curve was generated to assess the diagnostic ability of miRNA-33a-5p in LUSC and normal lung tissues based on in-house RT-qPCR. AUC: area under the curve; CI: confidence interval; LUSC: lung squamous cell carcinoma; ROC: receiver operating characteristic; RT-qPCR: quantitative real-time PCR.

The expression profile of miRNA-33a-5p examined from the 20 paired LUSC and normal lung tissues via in-house RT-qPCR showed that miRNA-33a-5p expression was prominently higher in the LUSC tissues than in the normal lung tissues (1.55 ± 0.13 vs. 0.98 ± 0.11, *P* = 0.001; Figure 1(a), Table 1). The AUC of the ROC curve based on RT-qPCR data was 0.7950 (*P* = 0.0014, Figure 1(b)), suggesting that miRNA-33a-5p has a moderate capability to distinguish the LUSC samples from the adjacent normal lung specimens. However, no statistically significantly difference was found regarding the relevance between miRNA-33a-5p expression level and other clinical factors (all *P* values > 0.05, Table 1).

### 3.2. miRNA-33a-5p Expression in LUSC Based on Public Online Databases

**Figure 2:**
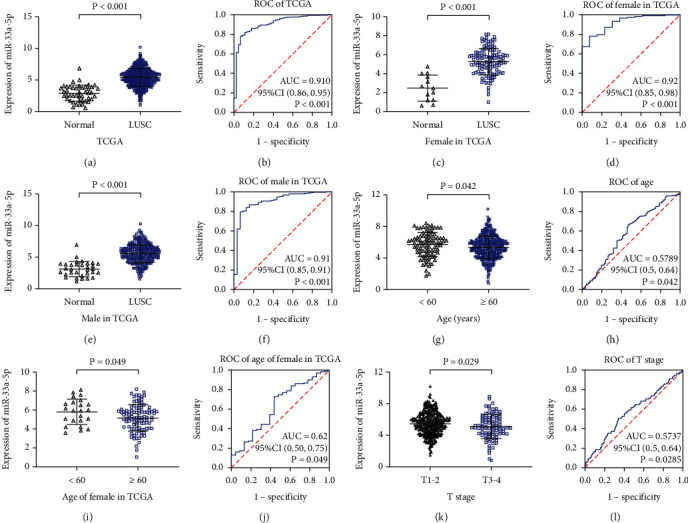
miRNA-33a-5p overexpression in LUSC and its association with clinicopathological parameters based on TCGA. (a) The expression of miRNA-33a-5p in LUSC and nontumor tissues based on TCGA. (b) The ROC curve was generated to assess the diagnostic ability of miRNA-33a-5p in LUSC and noncancerous lung tissues. (c) The expression of miRNA-33a-5p in LUSC and nontumor tissues for female patients based on TCGA. (d) The ROC curve of miRNA-33a-5p expression in female patients based on TCGA. (e) The expression of miRNA-33a-5p in LUSC and nontumor tissues for male patients based on TCGA. (f) The ROC curve of miRNA-33a-5p expression in male patients based on TCGA. (g) The expression of miRNA-33a-5p for age of LUSC patients. (h) The ROC curve of miRNA-33a-5p for age of LUSC. (i) The expression of miRNA-33a-5p in LUSC and nontumor tissues for different age of female patients based on TCGA. (j) The ROC curve of miRNA-33a-5p expression in different age of female patients based on TCGA. (k) The expression of miRNA-33a-5p in early (T1–T2) and late (T3–T4) T stages of LUSC. (l) The ROC curve of miRNA-33a-5p for T stages of LUSC. AUC: area under the curve; CI: confidence interval; LUSC: lung squamous cell carcinoma; ROC: receiver operating characteristic; TCGA: The Cancer Genome Atlas.

To confirm the analytic results of in-house RT-qPCR experiment, the miRNA-seq data of 478 LUSC samples and 45 adjacent non-LUSC specimens were simultaneously downloaded from the TCGA website. miRNA-33a-5p expression was presented in the form of mean ± SD. The results indicated that miRNA-33a-5p was clearly overexpressed in the LUSC group compared to the noncancerous group (5.42 ± 1.42 vs. 2.89 ± 1.26, *P* < 0.001; Figure 2(a)). The AUC was 0.910 (*P* < 0.001) suggested that miRNA-33a-5p was favorably able to differentiate the LUSC samples from non-LUSC samples (Figure 2(b)). After adjusting the data of Figure 2(a) for sex, we could find that miRNA-33a-5p also was significantly overexpressed in both female and male LUSC patients compared with normal (Figures 2(c)–2(f)), which indicated that the overexpression of miRNA-33a-5p in LUSC was not related to the gender. Compared to LUSC patients with age < 60 (5.71 ± 1.53), the expression of miRNA-33a-5p in LUSC patients with age ≥ 60 (5.36 ± 1.38) was notably downregulated (*P* = 0.042, Figures 2(g)–2(h)). After adjusting the data of Figure 1(i) for sex, the expression of miRNA-33a-5p was also upregulated in female patients with age < 60 (Figures 2(i) and 2(j)), however, the similar result was not found in male patients (data not shown). Patients with early T stage (T1-2) exhibited higher miRNA-33a-5p expression (5.49 ± 1.39) than patients with advanced T stage (T3-4) (5.11 ± 1.52) (*P* = 0.029, Figures 2(k) and 2(l)). However, no statistically significantly difference was found regarding the relevance between miRNA-33a-5p expression level and other clinical features (all *P* values > 0.05, Table 2).

**Figure 3:**
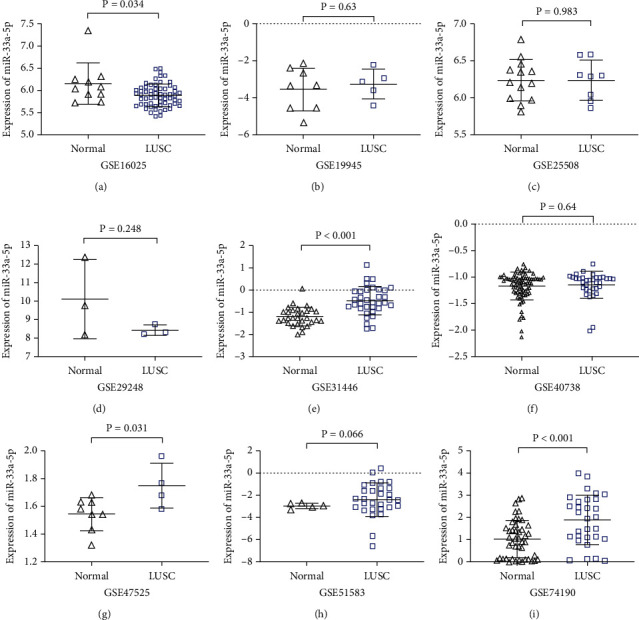
miRNA-33a-5p expression in LUSC and nontumor tissues based on miRNA microarrays. The scatter plots display the differential expression levels of miRNA-33a-5p in LUSC and noncancer tissues for each of the included microarray datasets. Data are expressed as the means ± SD, and *P* < 0.05 indicates a statistically significant difference when compared to the normal control. LUSC: lung squamous cell carcinoma.

**Figure 4:**
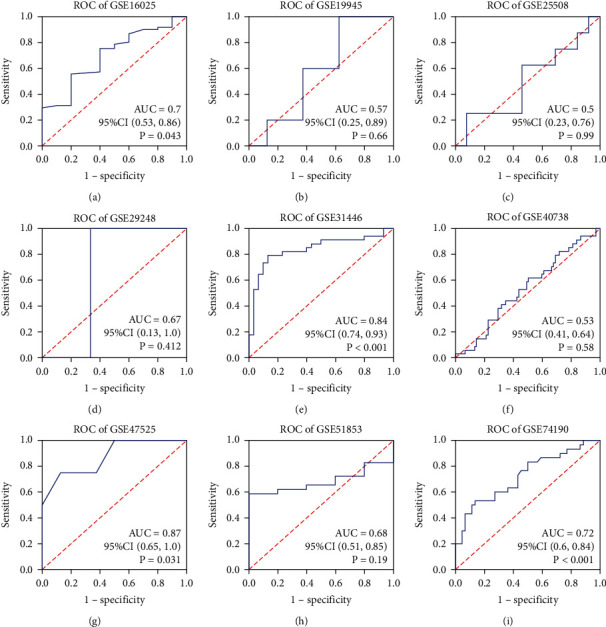
ROC curves based on miRNA microarray datasets. A panel of ROC curves shows the diagnostic ability of miRNA-33a-5p for LUSC in each of the included miRNA microarray datasets. AUC: 0.5–0.7 (low), 0.7–0.9 (moderate), and 0.9–1.0 (high). *P* < 0.05 indicates a statistically significant difference. ROC: receiver operating characteristic; AUC: area under the curve; CI: confidence interval.

With regard to miRNA microarrays, a total of nine microarray datasets (GSE16025, GSE19945, GSE25508, GSE29248, GSE40738, GSE47525, GSE51853, GSE56036, and GSE74190) obtained from GEO were included in the current study. The expression profile of miRNA-33a-5p and matching ROC curve in each microarray are shown in Figures 3 and 4, respectively. In these nine included datasets, only two datasets (GSE16025 and GSE40738) contained the clinical parameters of age and gender; then we evaluated the miRNA-33a-5p expression by stratifying for age and gender in these two datasets. The results showed that the expression of miRNA-33a-5p was not significantly different between LUSC and non-LUSC tissues in different age and sex groups in GSE16025 and GSE40738 datasets (Supplementary Figure 1).

### 3.3. Integrated Analysis Combining RT-qPCR, TCGA, and the Microarray Datasets

**Figure 5:**
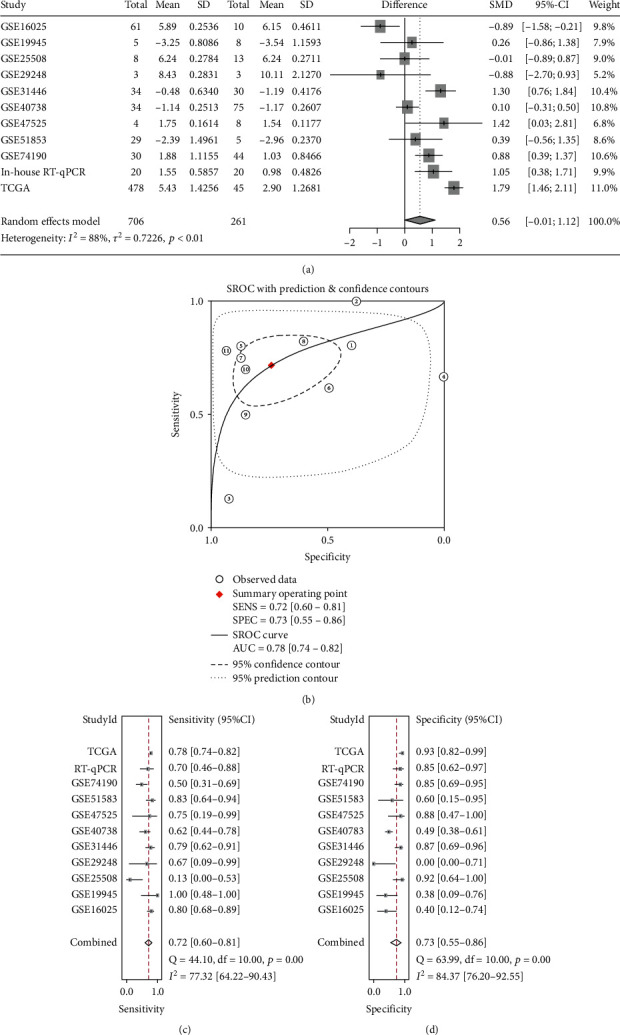
Integrated analysis of the miRNA-33a-5p expression value obtained from RT-qPCR, TCGA dataset, and public miRNA microarrays. (a) Forest plot of SMD to validate the high expression of miRNA-33a-5p in LUSC. (b) The SROC curve of miRNA-33a-5p in the diagnosis ability of LUSC data from all involved datasets (AUC = 0.78, 95% CI: 0.74-0.82). (c) The forest plot of sensitivity; (d) The forest plot of specificity. *P* < 0.05 indicates a statistically significant difference. SMD: standard mean difference; TCGA: The Cancer Genome Atlas; CI: confidence interval; SROC: summary receiver operating characteristic; AUC: area under the curve; RT-qPCR: quantitative real-time PCR.

To be more accurately evaluated the expression status of miRNA-33a-5p in LUSC, a comprehensive analysis was conducted integrating data from RT-qPCR, TCGA, and the microarray datasets. A random effects model was applied given the computed *I*^2^ = 88%, which might be due to the differentiation among the patients, the methods used to obtain different samples, or the statistical approaches used to analyze the data. The pooled SMD was 0.56 (*P* < 0.01, 95% CI: -0.01, 1.12) suggesting that miRNA-33a-5p overall overexpression in LUSC tissues (Figure 5(a)). SROC curve and forest plots of sensitivity (SEN) and specificity (SPE) supported the power of miRNA-33a-5p in distinguishing LUSC from noncancer tissues (AUC = 0.78, 95% CI: 0.74-0.83, SEN = 0.72, SPE = 0.73) (Figures 5(b)–5(d)).

### 3.4. Prognosis Value Analysis of miRNA-33a-5p in LUSC

**Figure 6:**
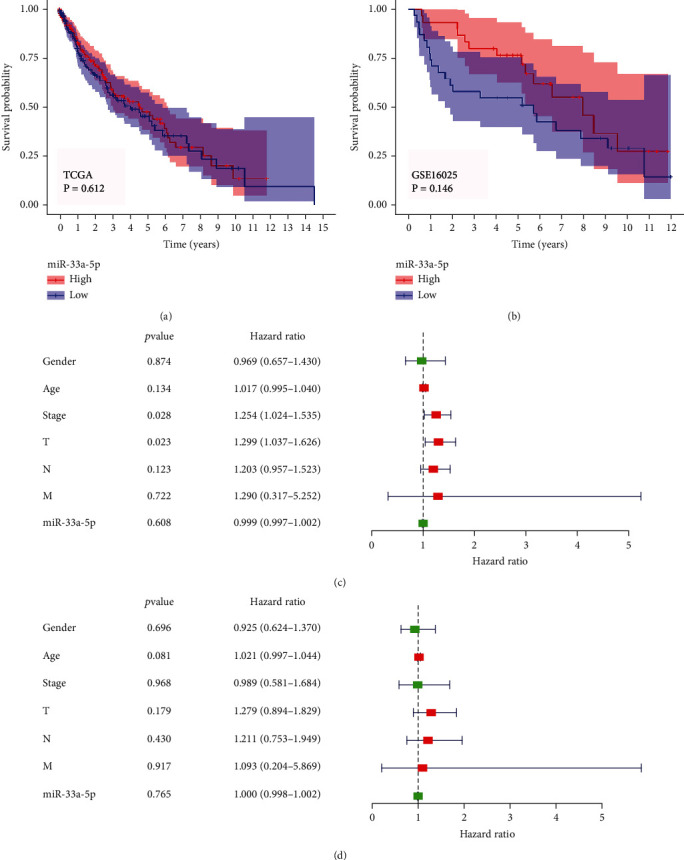
Prognostic analysis of miRNA-33a-5p in LUSC. (a, b) Survival analysis of miRNA-33a-5p based on TCGA and GSE16025, respectively. (c, d) Cox regression analysis for the prognostic value of miRNA-33a-5p and LUSC clinicopathological parameters according to univariate Cox and multivariate Cox regression analysis, respectively. *P* < 0.05 indicated statistically significant difference. TCGA: The Cancer Genome Atlas; T: tumor; N: node; M: metastasis.

There was no significant difference between overall survival outcomes of LUSC patients with high or low miRNA-33a-5p expression based on TCGA and GSE16025 datasets (Figures 6(a) and 6(b)). The results for the univariate Cox analysis suggested that T stage and pathological stage were the two risk factors for patients with LUSC (Figure 6(c)). However, there were no independent prognostic factors for predicting the prognosis of patients with LUSC according to multivariate Cox analysis (Figure 6(d)).

### 3.5. Screening the Target mRNAs of miRNA-33a-5p

**Figure 7:**
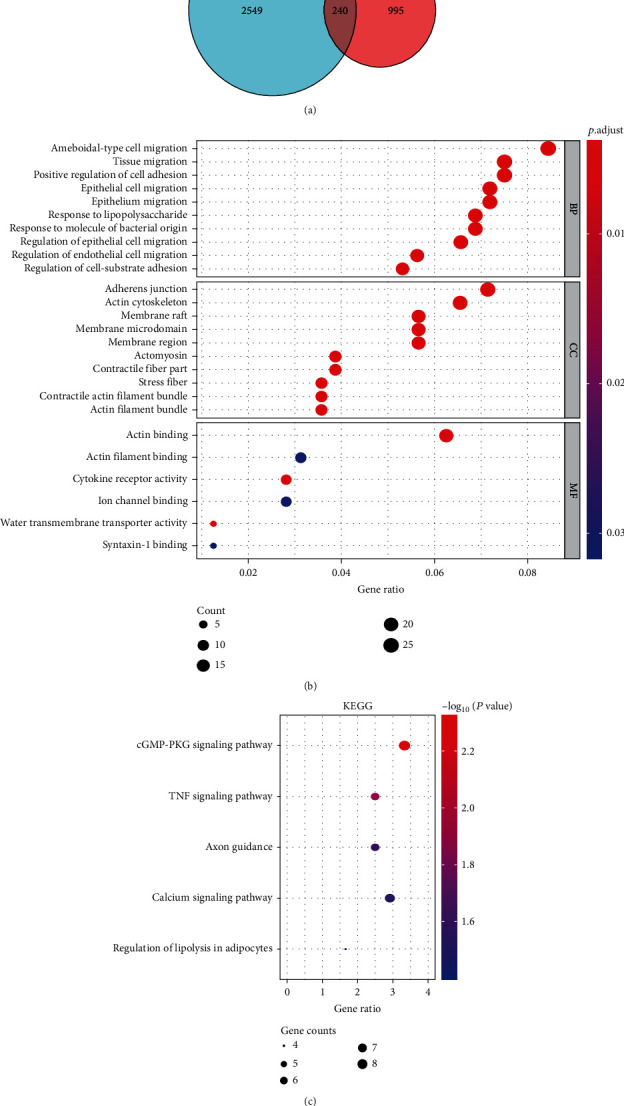
Biological functions analyses of the predicted target genes of miRNA-33a-5p. (a) Venn diagram of overlapping genes from the intersection of two independent datasets. (b) GO enrichment analysis bubble diagram. (c) KEGG pathway enrichment analysis bubble diagram. The *x*-axis represents the numbers of involved genes, and the *y*-axis represents the GO and KEGG terms. Each bubble represents a term. The size of the bubble indicates the number of involved genes. Red indicates higher degrees of significance of gene enrichment analysis than blue. DEGs: differentially expressed genes; GO: Gene Ontology; KEGG: Kyoto Encyclopedia of Genes and Genomes; BP: biological process; CC: cellular component; MF: molecular function.

Using the 12 miRNA platforms that were previously mentioned, 2,789 candidate target genes of miRNA-33a-5p were identified. Moreover, 1,235 downregulated DEGs in LUSC samples were derived from RNA-seq and miRNA microarrays. After overlapping these 1,235 downregulated DEGS with the 2,789 candidate targets of miRNA-33a-5p, 240 genes were identified as predicted target genes of miRNA-33a-5p (Figure 7(a)).

### 3.6. Functional Enrichment Analysis and the PPI Network

The previously mentioned 240 overlapping mRNAs were utilized for functional enrichment analyses to investigate their underlying molecular mechanism in LUSC. In GO function analysis, the significant enrichment terms of biological processes (BP), cell components (CC), and molecular functions (MF) are displayed in Figure 7(b). The KEGG analysis results suggest that these target genes were mostly significantly enriched in cGMP-PKG signaling pathway, TNF signaling pathway, Axon guidance, and calcium signaling pathway (Figure 7(c)).

**Figure 8:**
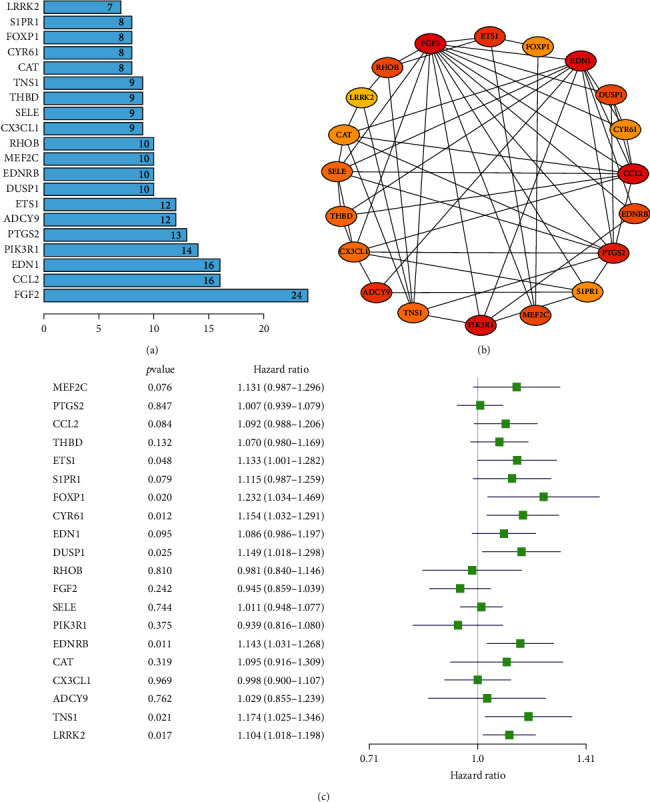
PPI network and hub gene analysis. (a) Histogram of top 20 key genes. The *y*-axis represents the name of genes, the *x*-axis represents the number of adjacent genes, and height is the number of gene connections. (b) PPI network of top 20 key genes. (c) Cox univariate regression analysis of top 20 hub genes. *P* < 0.05 indicated statistically significant difference.

Furthermore, a PPI network was constructed on STRING online tool using 240 target genes of miRNA-33a-5p. The importance of the protein in the PPI network depends on its connection and the number of occurrences in the network. We statistically analyzed the number of protein occurrences in the network and listed the top 20 genes that appeared most frequently (Figure 8(a)). Then, we imported these 20 genes into Cytoscape v3.7.1 to obtain a novel subnetwork (Figure 8(b)). Subsequently, we applied a univariate Cox regression analysis to explore the prognostic capabilities of these 20 genes in LUSC patients. According to the univariate Cox regression analysis, ETS1, CYR61, DUSP1, EDNRB, TNS1, FOXP1, and LRRK2 were the genes with prognostic ability (*P* < 0.05, Figure 8(c)).

**Figure 9:**
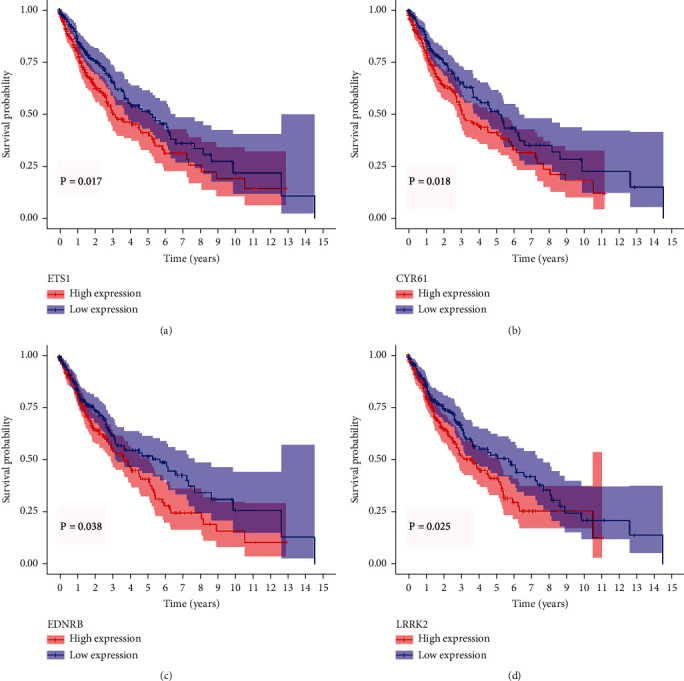
Survival analysis of four prognosis-related hub target genes of miRNA-33a-5p based on TCGA dataset. (a) ETS1. (b) CYR61. (c) EDNRB. (d) LRRK2. *P* < 0.05 indicates a statistically significant difference.

According to the multivariate Cox analysis, all the seven prognosis-related genes could not be used as independent prognostic factors for predicting the prognosis of patients with LUSC (Supplementary Figure 2). Moreover, survival analyses were carried out to evaluate the overall survival outcome of the above-mentioned seven prognosis-related genes in LUSC. Among these seven genes, overexpression of four genes (ETS1, CYR61, EDNRB, and LRRK2) were related to a poorer overall survival status in LUSC (*P* < 0.05, Figure 9). Finally, ETS1, CYR61, EDNRB, and LRRK2 were chose as core target miRNAs of miRNA-33a-5p in our study. According to the multivariate Cox regression analysis, RHOB and FGF2 were the genes with prognostic ability (*P* < 0.05, Supplementary Figure 2). The survival analyses were also carried out to evaluate the overall survival outcome of these two prognosis-related genes in LUSC. The results indicated that overexpression of these two genes did not play a vital role in the poorer prognosis in LUSC (*P* > 0.05, Supplementary Figure 3).

### 3.7. Clinical Expression of Hub Target Genes of miRNA-33a-5p

**Figure 10:**
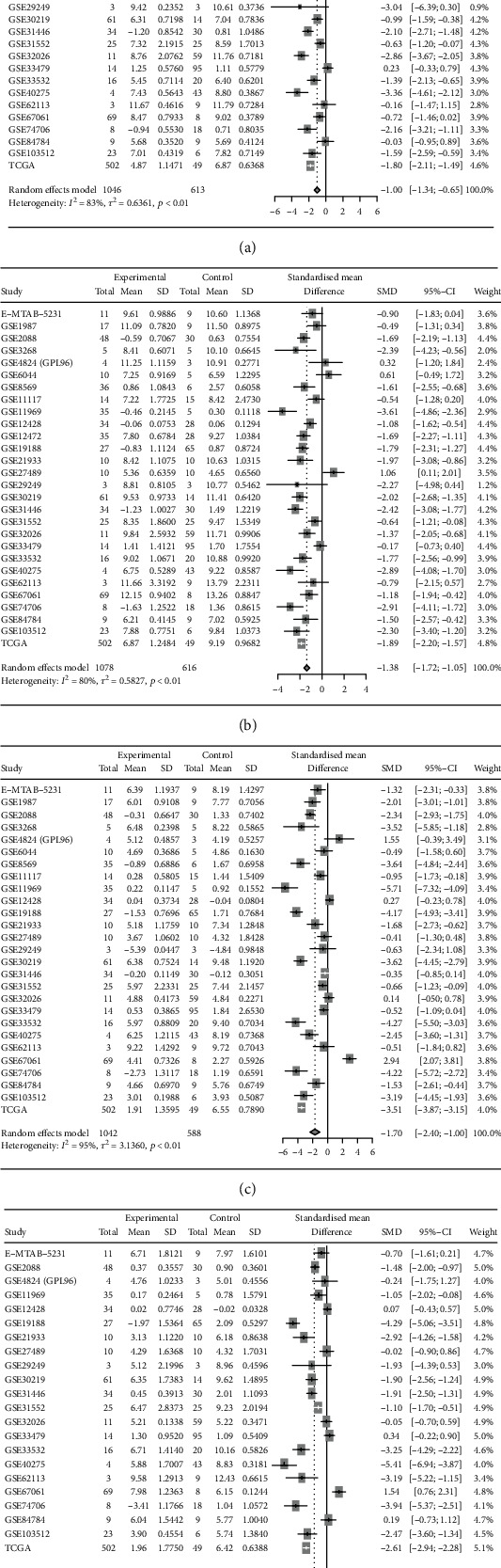
The integrated SMD of four prognosis-related hub genes expression in LUSC. (a-d) Forest plots of SMD for ETS1, CYR61, EDNRB, and LRRK2, respectively. SD: standard deviation; SMD: standardized mean difference; CI: confidence interval.

Aim to validate the expression status of four hub target genes (ETS1, CYR61, EDNRB, and LRRK2) in LUSC, we also comprehensively integrated analysis the expression data of these genes from RNA-seq and miRNA microarrays. The pooled SMD of ETS1, CYR61, EDNRB, and LRRK2 were -1.00, -1.38, -1.70, and -1.58, respectively (Figure 10), which illustrated that the expression of these genes notably down-regulated in LUSC.

**Figure 11:**
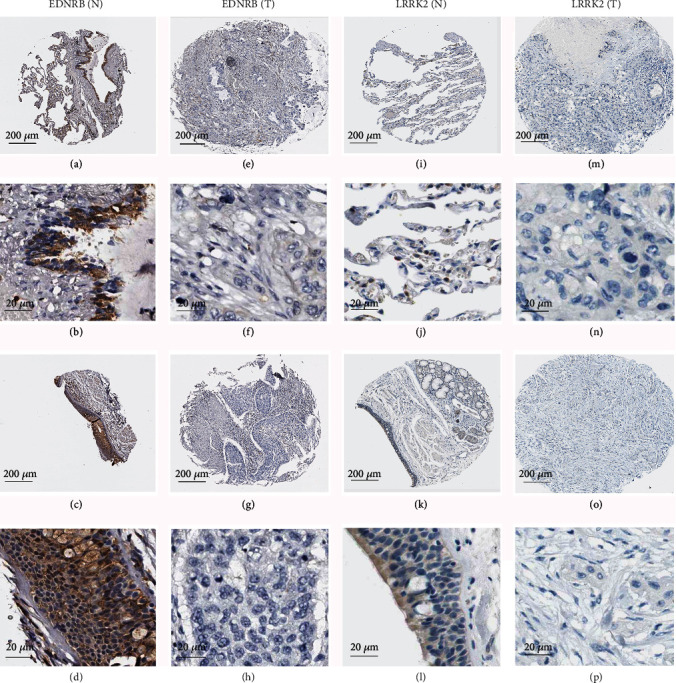
Protein expression of two prognosis-related hub genes in the normal lung and LUSC tissue based on The Human Protein Atlas database. (a, b) EDNRB protein expression level in normal lung was low (intensity: low, quantity: 25-50%, location: cytoplasmic/membranous). Antibody HPA027546 was used. (c, d) EDNRB protein expression level in a normal bronchus tissue was moderate (intensity: moderate, quantity: 50-75%, location: cytoplasmic/membranous). Antibody HPA027546 was used. (e–h) EDNRB protein expression level in LUSC tissue was not detected (intensity: negative, quantity: none, location: none). Antibody HPA027546 was used. (i, j) LRRK2 protein expression level in normal lung was moderate (intensity: moderate, quantity: 25-75%, location: cytoplasmic/membranous). Antibody HPA014293 was used. (k, l) LRRK2 protein expression level in normal bronchus tissue was low (intensity: low, quantity: 25-50%, location: cytoplasmic/membranous). Antibody HPA014293 was used. (m–p) LRRK2 protein expression level in LUSC tissue was not detected (intensity: negative, quantity: none, location: none). Antibody HPA014293 was used. Note: N: normal; T: tumor.

**Figure 12:**
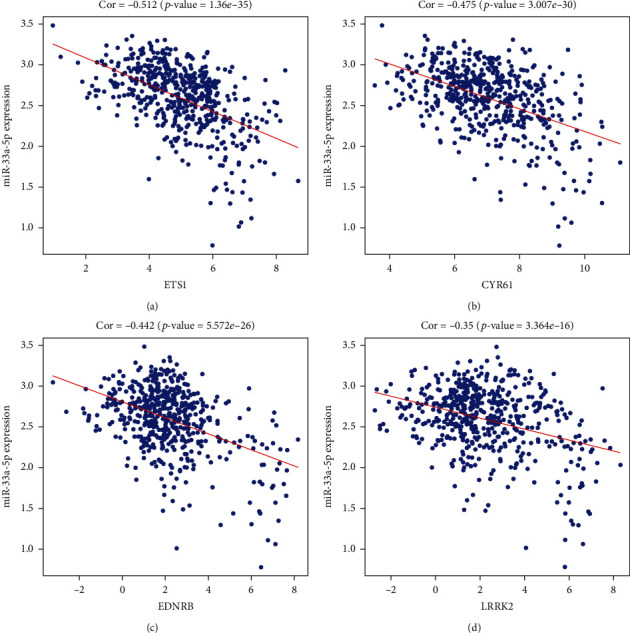
Pearson's correlation analysis of miRNA-33a-5p expression and the expression of the four prognosis-related hub genes. (a–d) Correlations between miRNA-33a-5p and ETS1, CYR61, EDNRB, and LRRK2, respectively. *P* < 0.05 indicated statistical significance.

Apart from investigating the mRNA level of four key target genes, the protein levels were also explored through The Human Protein Atlas database (THPA) database. However, only two genes' (EDNRB and LRRK2) protein expression levels were consistent with mRNA expression, and they were all downregulated in LUSC tissues (Figure 11).  Figure 12 elucidates that four core target genes' expression levels were all remarkably negatively correlated with the expression of miRNA-33a-5p.

## 4. Discussion

Considering the vital role of miRNAs in tumor, several previous researches have described the expression level and specific mechanism of miRNA-33a-5p in LC [22–25]. However, no relevant studies in the literature concerned the specific role of miRNA-33a-5p in the occurrence and evolution of LUSC. As far as we know, we are the first group to thoroughly study the expression level, clinicopathological value, and underlying biological mechanism of miRNA-33a-5p in LUSC.

One of the highlights in our study is that we adopted various methods and gathered large a number of cases to uncover the expression profile and relevant role of miRNA-33a-5p in LUSC. miRNA-33a-5p overexpressed in LUSC was supported by 706 LUSC and 261 non-LUSC samples gathering from RT-qPCR, miRNA-seq, and public miRNA microarrays in present study. Upregulation of miRNA-33a-5p may suggest that it could play a pivotal role in the occurrence and progression of LUSC as an oncogene. Interestingly, miRNA-33a-5p expression was clearly downregulated in NSCLC and LUAD based on some previous studies [22, 23, 25, 26], which contrast with the result of our study. Therefore, we could conclude that miRNA-33a-5p plays a specific important role in LUSC compared with NSCLC and LUAD. Furthermore, upregulation of miRNA-33a-5p in LUSC was dramatically relevant to age < 60 years and early T stage, indicating that age and T stage may affect the differential expression of miRNA-33a-5p in LUSC. Regrettably, no significant difference was found in overall survival of LUSC patients with different expression level of miRNA-33a-5p, and it could not act as an independent prognostic factor in LUSC based on prognosis analysis in our study.

After proving the upregulation and oncogenic function of miRNA-33a-5p in LUSC, we further explored the underlying molecular mechanism of miRNA-33a-5p in LUSC through functional enrichment analysis of 240 predicted target genes. According to KEGG enrichment analysis, these target genes were prominently clustered in pathways including the cGMP-PKG signaling pathway, TNF signaling pathway, Axon guidance, calcium signaling pathway, and regulation of lipolysis in adipocytes. Among the significantly enriched pathways, the cGMP-PKG signaling pathway and TNF signaling pathway played indispensable roles in the biological processes of human cancers [27]. Previous researches have verified that the activation of cGMP-PKG played a crucial role in controlling cellular *β*-catenin levels, and the latter was famous for its role in carcinogenesis, and it has been detected in several types of human malignant tumor, such as hepatocellular carcinoma [28], renal carcinoma [29], colon cancer [30], and prostate cancer [31]. A previous study reported that restraining cGMP-PKG pathway can decrease metastasis and invasion of breast cancer, because activation of PKG heightened the motility and infiltration of human breast carcinoma cells [32]. Gong et al. pointed out that propranolol can suppress cervical carcinoma cell proliferation by inhibiting the cGMP-PKG pathway [27]. The results of previous studies strongly support that the cGMP/PKG signaling pathway is closely related to the progression of malignance. Therefore, we speculate that miRNA-33a-5p may facilitate LUSC cell growth and invasion by activating cGMP-PKG pathway. However, this hypothesis requires further experimental validation in our study. Now, it has been confirmed that tumor necrosis factor (TNF, formerly referred to as TNF-alpha) can favor tumor growth and/or progression in vitro and vivo experiments [33–36]. Shih et al. [37] found out that TNF-alpha-380 A has a promotive role in the development and progression of LC. Unfortunately, the specific mechanism of the TNF signaling pathway in LUSC remains unclear. In view of the major role of TNF signaling pathways in malignancies, we considered that one of the carcinogenic mechanisms of miRNA-33a-5p in LUSC is accelerating the development and migration of LUSC cells by modulating the TNF signaling pathway.

In order to better understand the function of miRNA-33a-5p in LUSC, we predicted and analyzed the downstream target miRNAs of miRNA-33a-5p in LUSC. According to the PPI network, four prognosis-related downregulated DEGs (ETS1, EDNRB, CYR61, and LRRK2) were chosen as the final target genes of miRNA-33a-5p in LUSC. Transcription factor (TF) ETS1 participates in a variety of pathological and biological processes, such as oncogenesis, cell differentiation, apoptosis, and proliferation [38–40], and the aberrant expression of ETS1 has been proven to correlate with the development, invasion, and migration in various types of malignance, including breast cancer [41], prostate cancer [42], colorectal cancer [43], hepatocellular carcinoma [44], gastric cancer [45], and NSCLC [46]. However, few studies reported the specific molecular mechanism of ETS1 in LUSC as well as acting as a target gene of miRNA-33a-5p in LUSC. Our result indicated that ETS1 was prominently downregulated in LUSC and closely implicated with prognosis of LUSC patients, which strongly suggests that ETS1 may be a promising biomarker of LUSC and attractive target gene of miRNA-33a-5p in LUSC. Endothelin receptor type B (EDNRB) is usually underexpressed or even silenced by promoter hypermethylation in various human cancers by acting as an oncosuppressor [47–50]. In the field of LC, Wei et al. [51] uncovered that EDNRB acted as a prognostic factor for LUAD patients by regulating the ERK signaling pathway. However, there was no report of EDNRB in LUSC. A previous study revealed that EDNRB was mainly clustered in the cGMP-PKG signaling pathway [52], which is consistent with our result based on KEGG analysis. Hence, we infer that miRNA-33a-5p may facilitate the development and infiltration of LUSC through cGMP-PKG signaling pathway by targeting EDNRB. Of course, this hypothesis needs further experiment validation in our future study. Previous studies indicated that cysteine-rich angiogenic inducer 61 (CYR61) participated in regulating differentiation, cellular adhesion, mitogenesis, proliferation, survival, migration, invasion, and metastasis of tumor cell [53, 54]. Li et al. [55] discovered that upregulation of CYR61 played a pivotal role in the occurrence and progression of NSCLC cell via AKT and ERK signaling pathways. Hsu et al. [56] reported that downregulation of CYR61 could decrease progression of LC. Unfortunately, few researches play closely attention on the function of CYR61 in LUSC. Our study validated that downregulation of CYR61 was prominently associated with the prognosis of LUSC patient, but further studies are still needed to investigate the extract mechanism of miRNA-33a-5p in LUSC by targeting CYR61. A previous study revealed that leucine-rich repeat kinase 2 (LRRK2) was downregulated in LUSC and implicated with the overall survival rate for LUSC patients [57], which corresponded to our results. Fallaciously, the involved mechanism of LRRK2 in LUSC remains elusive until now. Further studies need to be done to confirm whether LRRK2 contributed to tumorigenesis or could be used as a therapeutic target in LUSC.

The study has several shortcomings. First, clinical sample size in the RT-qPCR experiment is small and lacking corresponding clinicopathological parameters of these samples in our study. Second, lacking in vitro and vivo experiment to confirm the relationship and implicated mechanism of miRNA-33a-5p and its target genes in LUSC. Third, in vitro and in vivo studies are needed to perform to determine mRNA targets and the functional role of miR-33a-5p in LUSC in our future work.

## 5. Conclusion

In our study, miRNA-33a-5p prominently overexpressed in LUSC was confirmed by integrating RT-qPCR, miRNA-seq, and miRNA microarrays, and it may act as an oncogene and promote the proliferation and migration of LUSC by modulating the cGMP-PKG signaling pathway and/or TNF signaling pathway. In summary, miRNA-33a-5p may contribute as a prospective therapeutic target in LUSC.

## Figures and Tables

**Table 1:** Associations between miRNA-33a-5p expression and clinicopathological features in LUSC based on RT-qPCR.

Clinicopathological feature	Category	*n*	Mean ± SD	*P* value
Tissue	Normal	20	0.97 ± 0.48	0.001^∗^
	LUSC	20	1.55 ± 0.58	
Age (years)	<60	15	1.73 ± 0.51	0.46
	≥60	5	1.55 ± 0.37	
Gender	Female	5	1.23 ± 0.43	0.19
	Male	15	1.65 ± 0.60	
Female	Normal	5	0.81 ± 0.42	0.26
	LUSC	5	1.23 ± 0.43	
Male	Normal	10	1.03 ± 0.50	0.04^∗^
	LUSC	10	1.65 ± 0.60	
Pathological stage	I-II	16	1.45 ± 0.39	0.39
	III-IV	4	1.66 ± 0.46	
T stage	T1-T2	18	1.50 ± 0.59	0.27
	T3-T4	2	1.99 ± 0.29	
Node	No	13	1.48 ± 0.32	0.49
	Yes	7	1.27 ± 0.44	

LUSC: lung squamous cell carcinoma: TCGA: The Cancer Genome Atlas; *n*: number; M: mean; SD: standard deviation. ^∗^*P* < 0.05 was considered statistically significant.

**Table 2:** Relationship between miRNA-33a-5p expression and clinicopathological parameters of LUSC from TCGA.

Clinicopathological feature		*n*	Mean ± SD	*P* value
Tissue	Normal	45	2.89 ± 1.26	<0.001^∗^
	LUSC	478	5.42 ± 1.42	
Age (years)	<60	87	5.71 ± 1.53	0.042^∗^
	≥60	391	5.36 ± 1.38	
Gender	Female	124	5.27 ± 1.39	0.234
	Male	354	5.47 ± 1.43	
Pathological stage	I-II	388	5.44 ± 1.41	0.498
	III-IV	88	5.35 ± 1.47	
T stage	T1-T2	387	5.49 ± 1.39	0.029^∗^
	T3-T4	91	5.11 ± 1.52	
Node	No	306	5.39 ± 1.39	0.497
	Yes	172	5.49 ± 1.47	
Metastasis	No	472	5.43 ± 1.42	0.516
	Yes	5	5.01 ± 1.99	
Tumor location	Peripheral	91	5.21 ± 1.34	0.419
	Central	140	5.31 ± 1.40	

LUSC: lung squamous cell carcinoma: TCGA: The Cancer Genome Atlas; *n*: number; M: mean; SD: standard deviation. ^∗^*P* < 0.05 was considered statistically significant.

## Data Availability

The data of miRNA-seq were downloaded from the website of TCGA database (https://tcga-data.nci.nih.gov/docs/publications/tcga/). The data of GSE datasets were obtained from the website of the GEO database (https://www.ncbi.nlm.nih.gov/geo/). The data of the First Affiliated Hospital of Guangxi Medical University cohort used to support the findings of this study are available from the corresponding author upon request.

## Supplementary Materials

Supplementary 1 Supplementary Figure 1: miRNA-33a-5p expression in different groups of age and gender based on GSE16025 and GSE40738 datasets. (a) The expression of miRNA-33a-5p in LUSC and nontumor tissues for female patients based on GSE16025. (b) The expression of miRNA-33a-5p in LUSC and nontumor tissues for male patients based on GSE16025. (c) The expression of miRNA-33a-5p in LUSC and nontumor tissues for patients with age < 60 based on GSE16025. (d) The expression of miRNA-33a-5p in LUSC and nontumor tissues for patients with age ≥ 60 based on GSE16025. (e) The expression of miRNA-33a-5p for the age of LUSC patients based on GSE16025. (f) The expression of miRNA-33a-5p for the gender of LUSC patients based on GSE16025. (g) The expression of miRNA-33a-5p in LUSC and nontumor tissues for female patients based on GSE40738. (h) The expression of miRNA-33a-5p in LUSC and nontumor tissues for male patients based on GSE40738. (i) The expression of miRNA-33a-5p in LUSC and nontumor tissues for patients with age < 60 based on GSE40738. (j) The expression of miRNA-33a-5p in LUSC and nontumor tissues for patients with age ≥ 60 based on GSE40738. (k) The expression of miRNA-33a-5p for the age of LUSC patients based on GSE40738. (l) The expression of miRNA-33a-5p for the gender of LUSC patients based on GSE40738. Data are expressed as the means ± SD, and *P* < 0.05 indicates a statistically significant difference when compared to the control. LUSC: lung squamous cell carcinoma.

Supplementary 2 Supplementary Figure 2: Cox multivariate regression analysis for the prognostic value of 7 prognosis-related genes and LUSC clinicopathological parameters according to TCGA. *P* < 0.05 indicates a statistical significance.

Supplementary 3 Supplementary Figure 3: survival analysis of two prognosis-related genes of miRNA-33a-5p based on TCGA dataset. (a) RHOB. (b) FGF2.
