# Supplementary figures and images for: Clinical Value and Potential Mechanism of miRNA-33a-5p in Lung Squamous Cell Carcinoma
Source: Anal Cell Pathol (Amst). 2021 Nov 29;2021:6614331. doi: 10.1155/2021/6614331 (PMC8649614; doi:10.1155/2021/6614331)

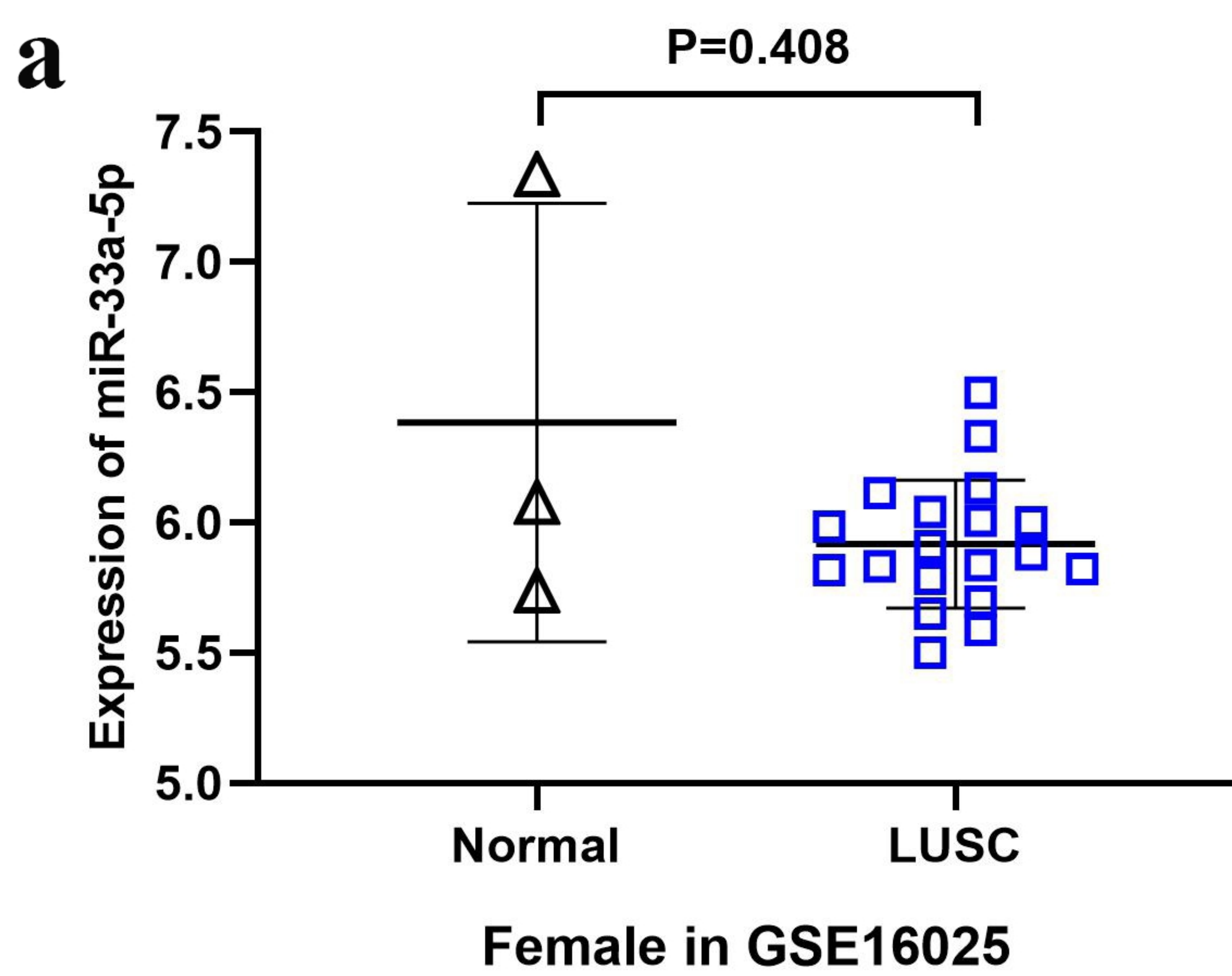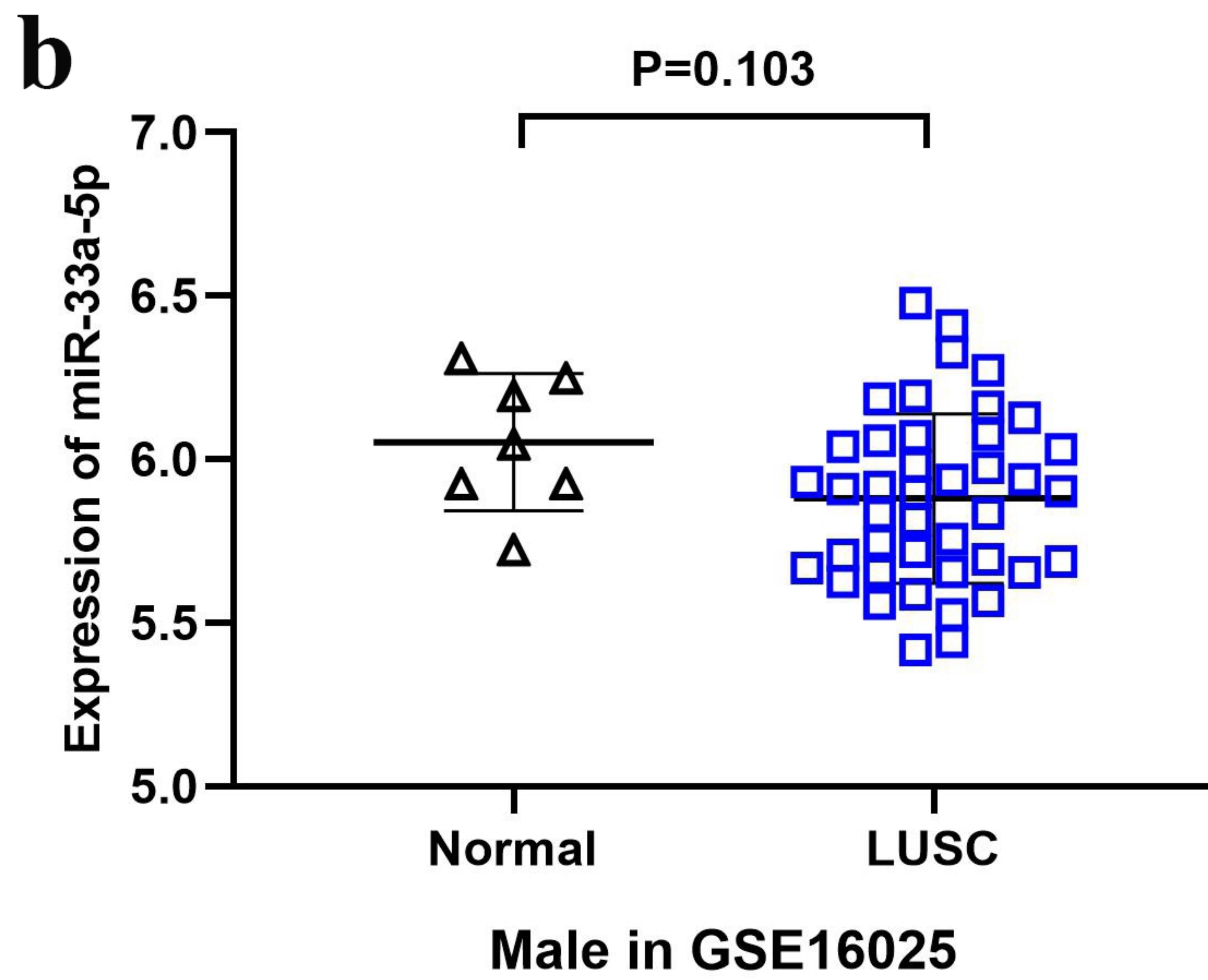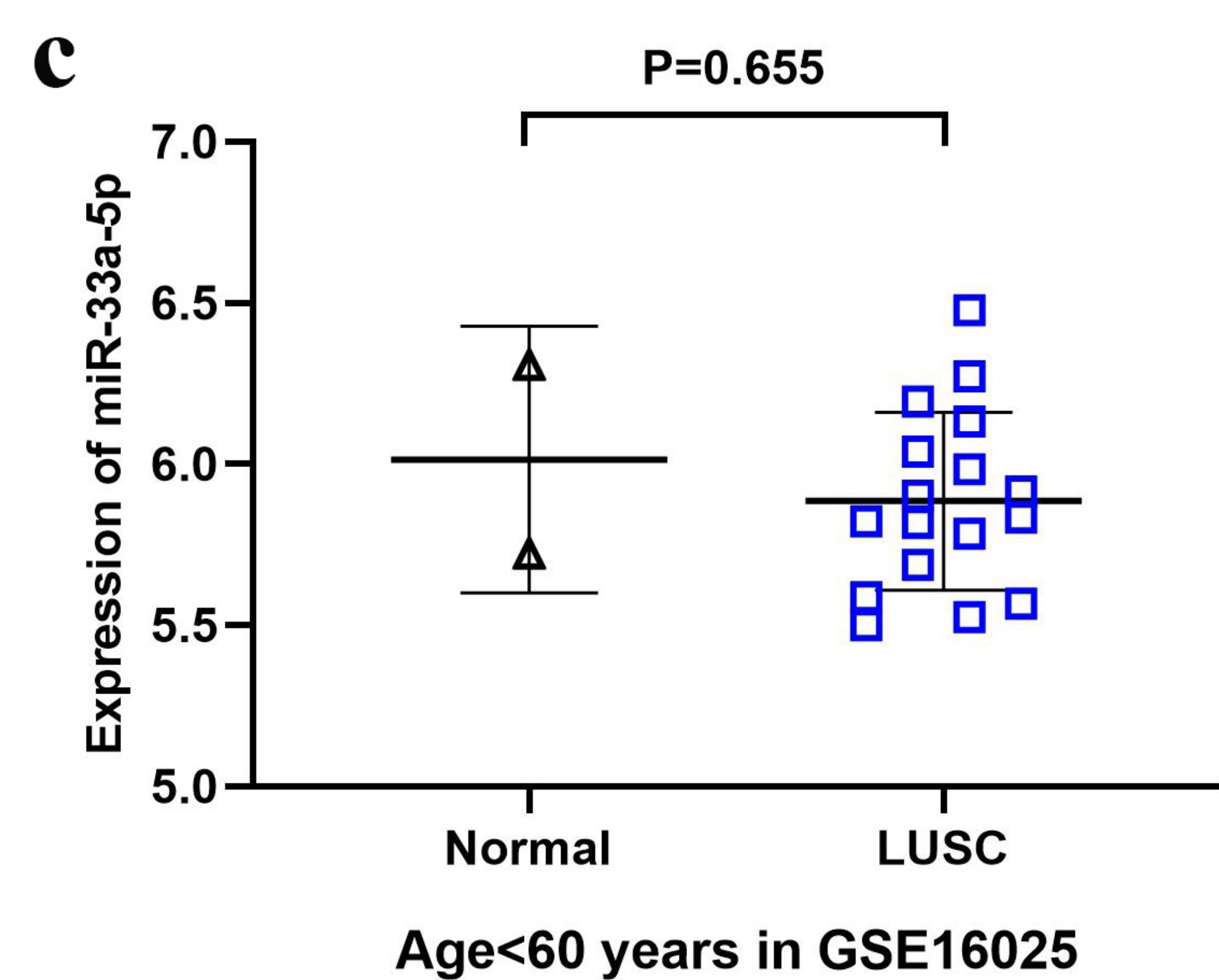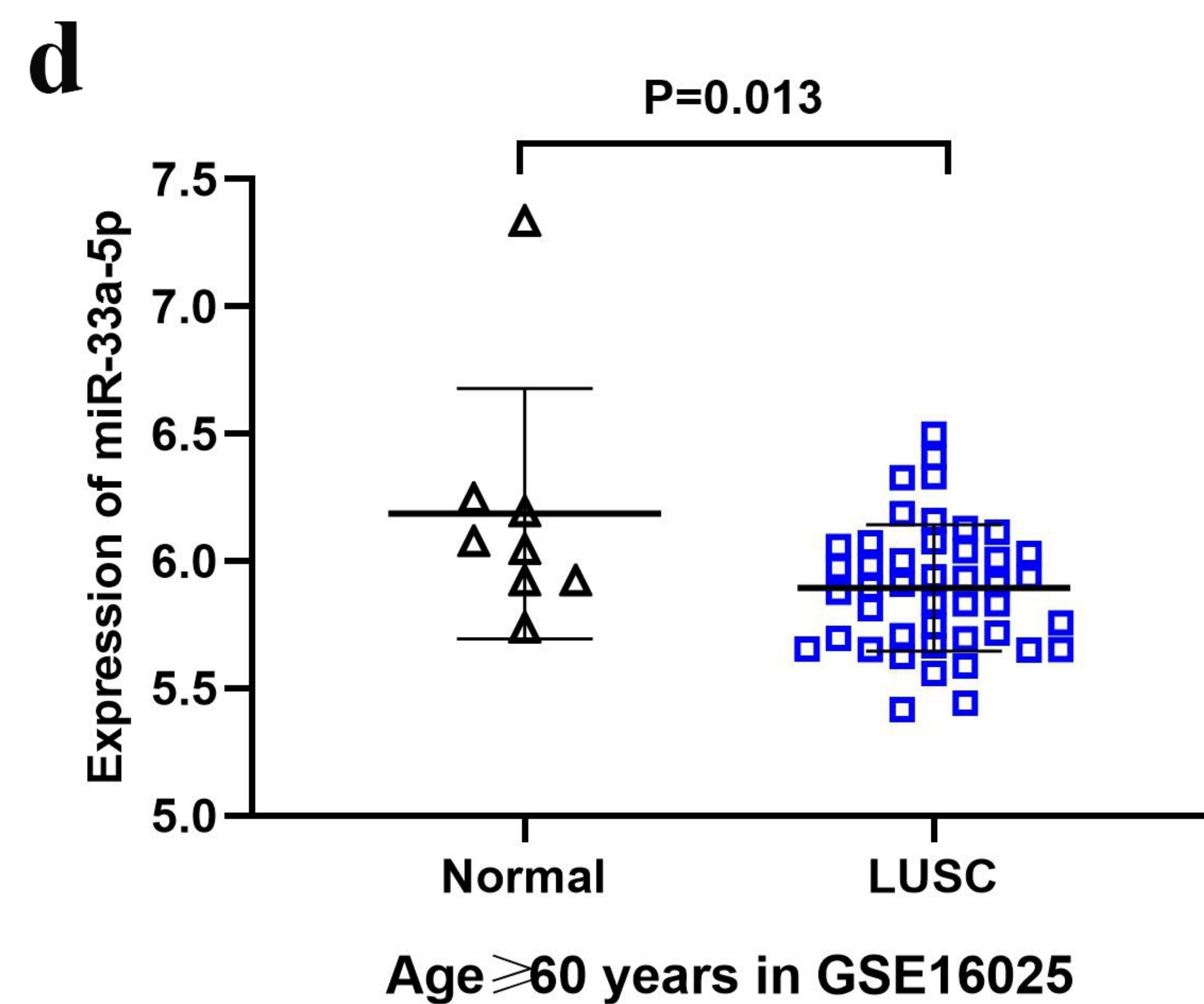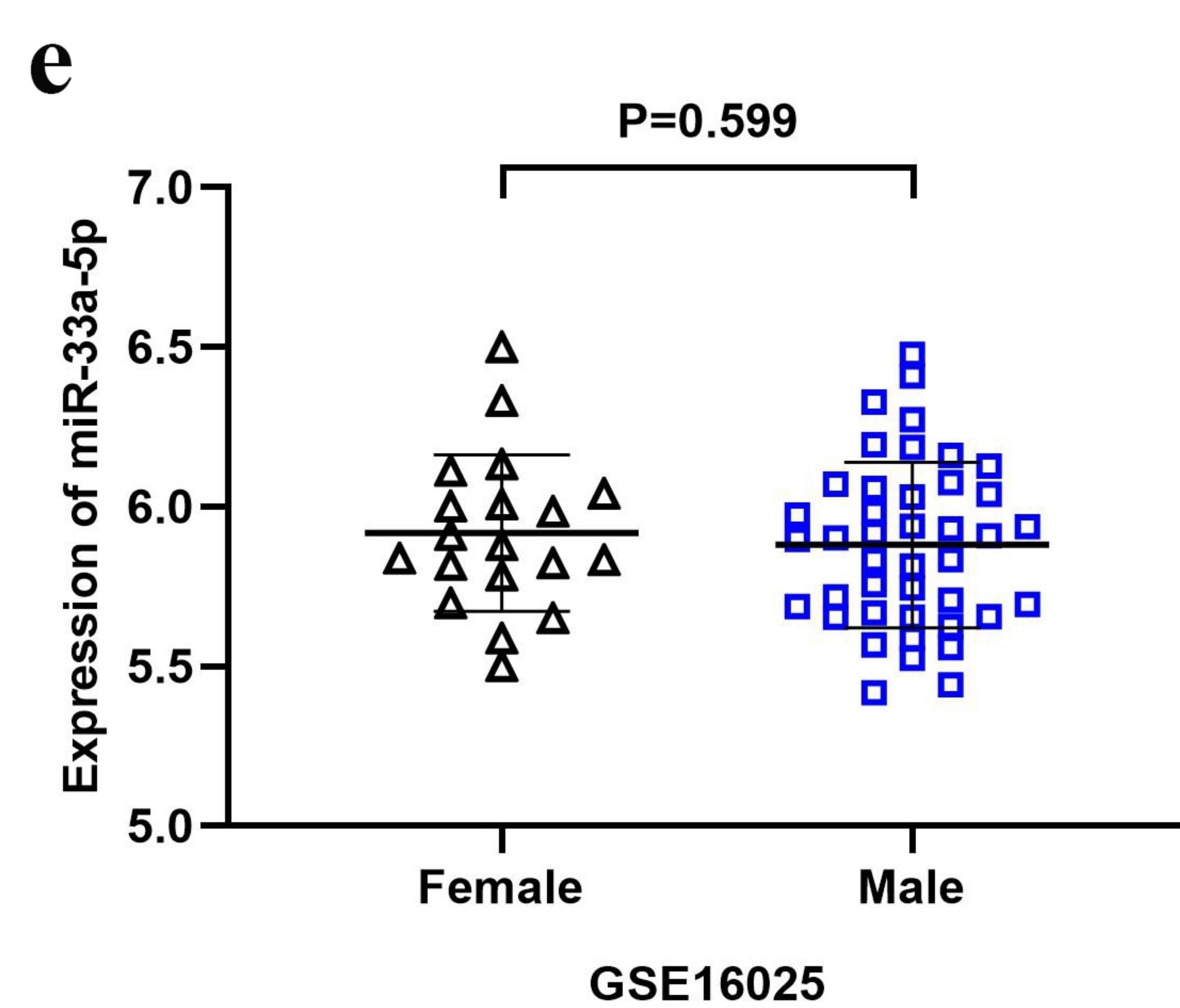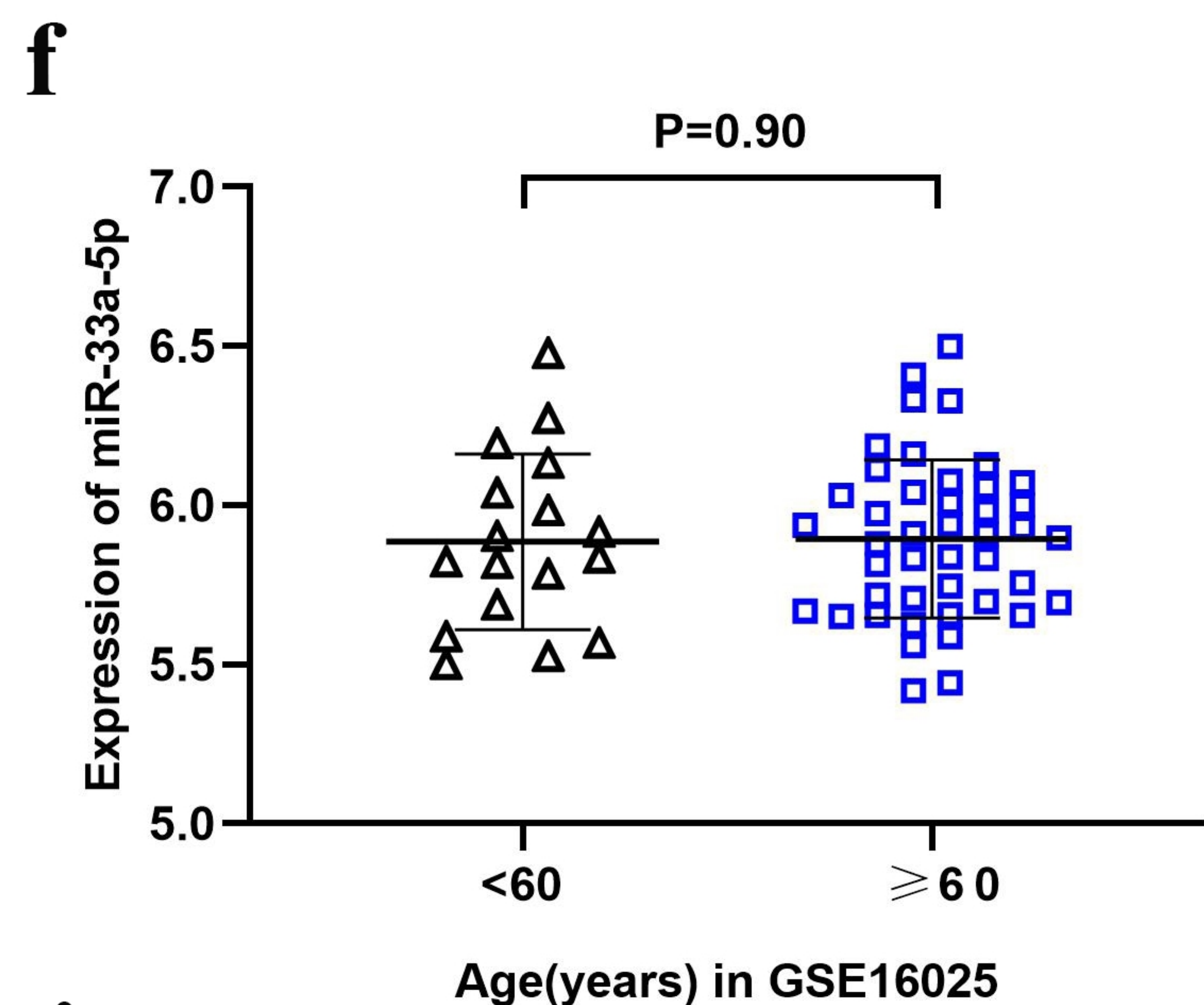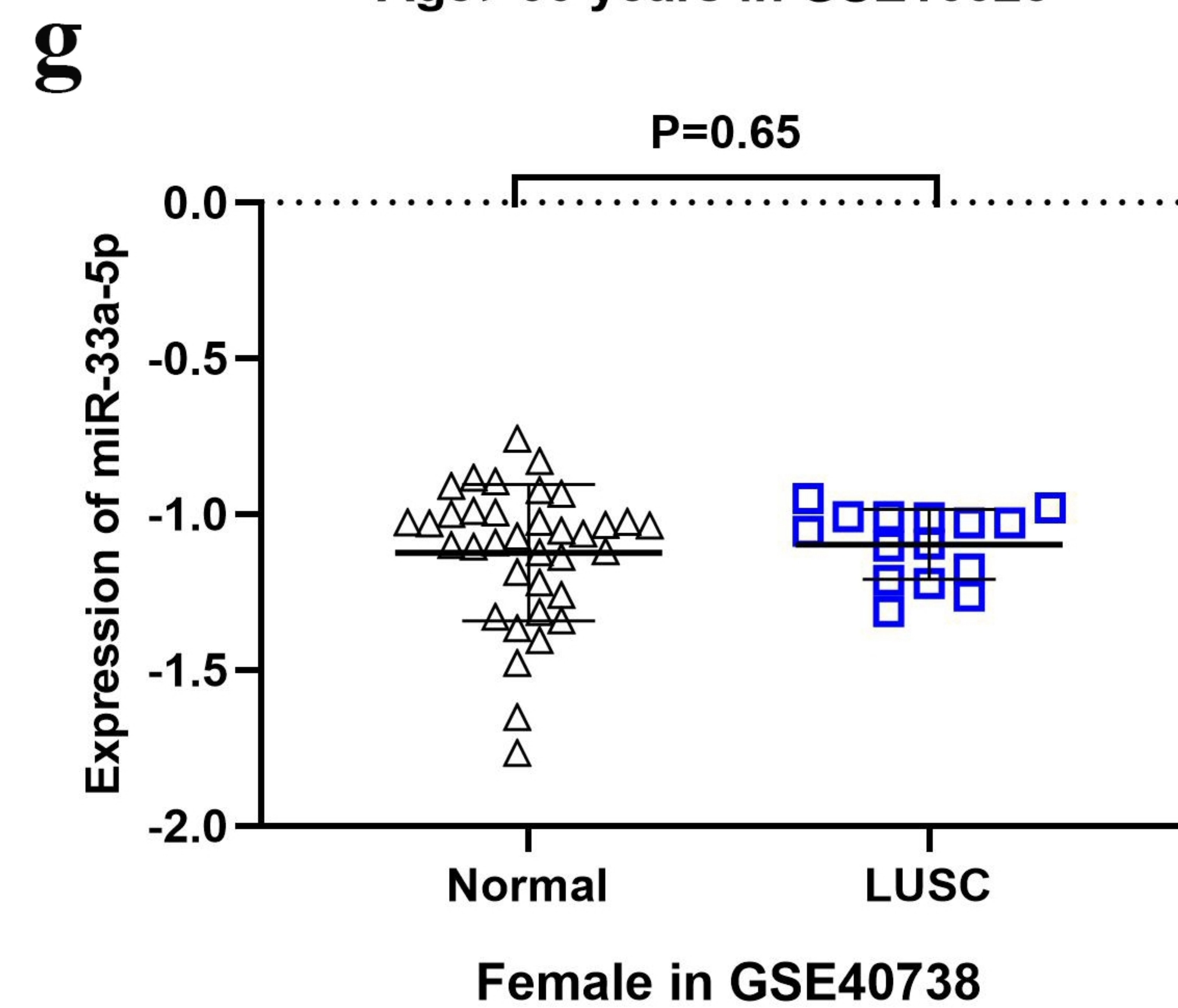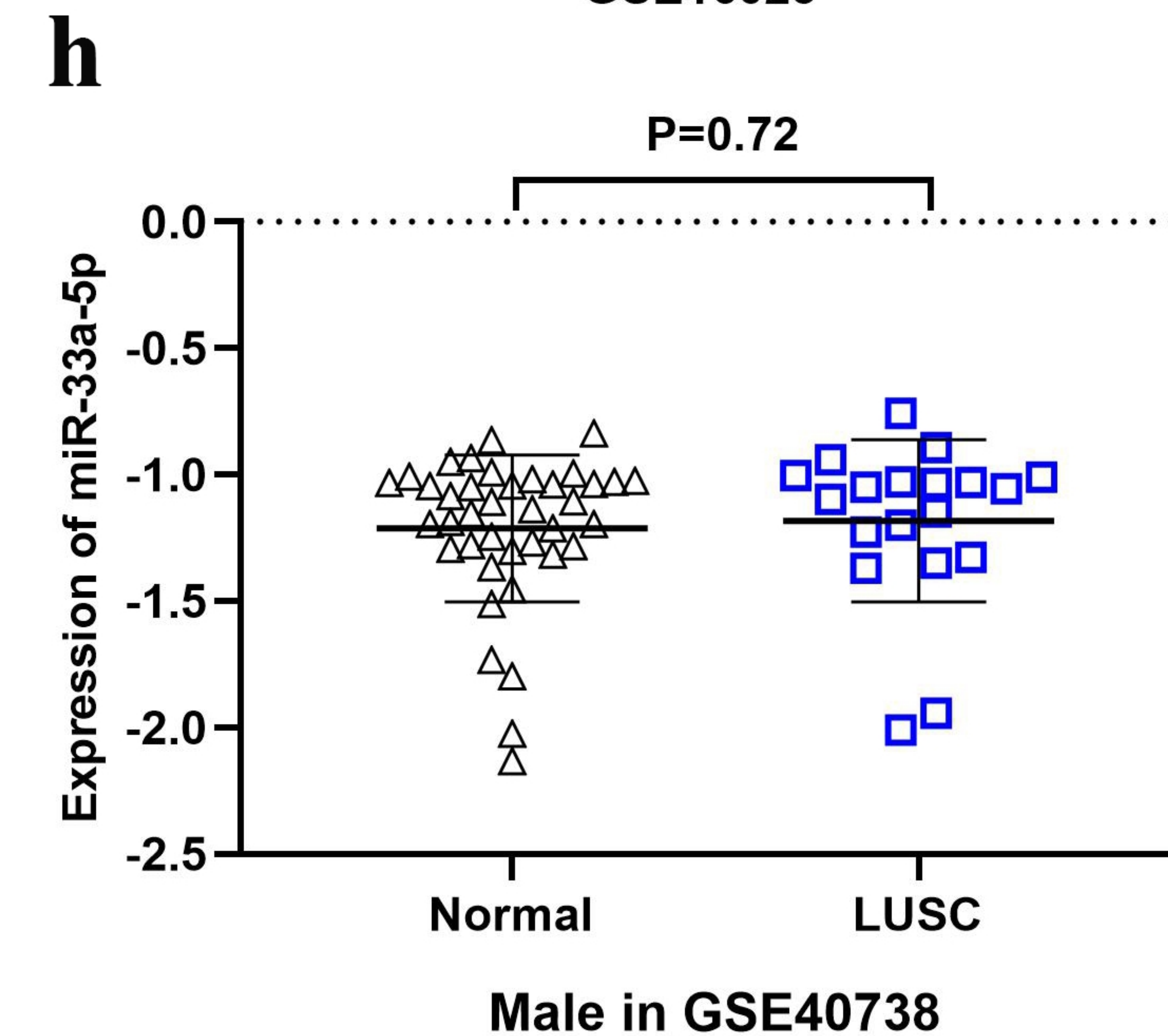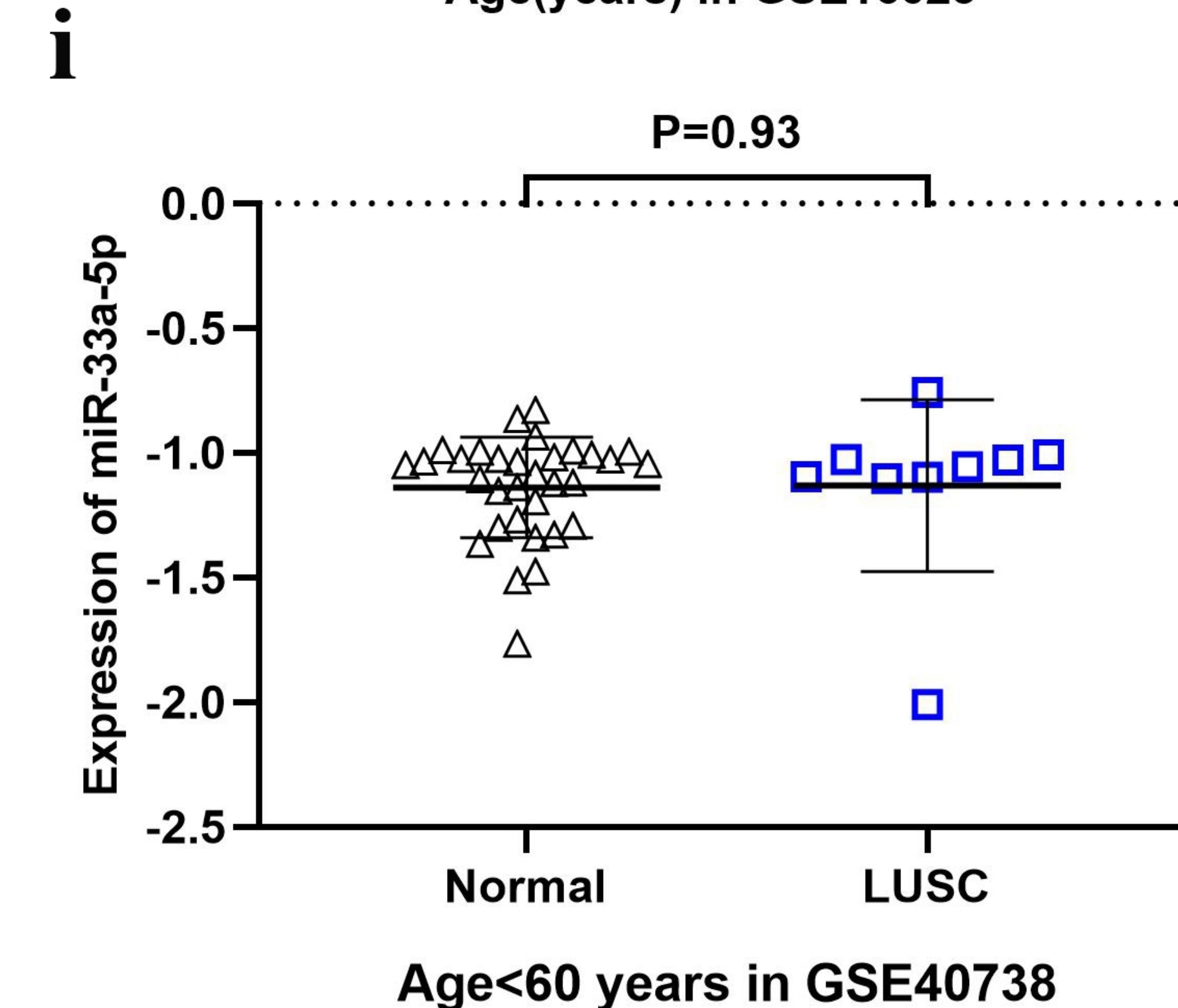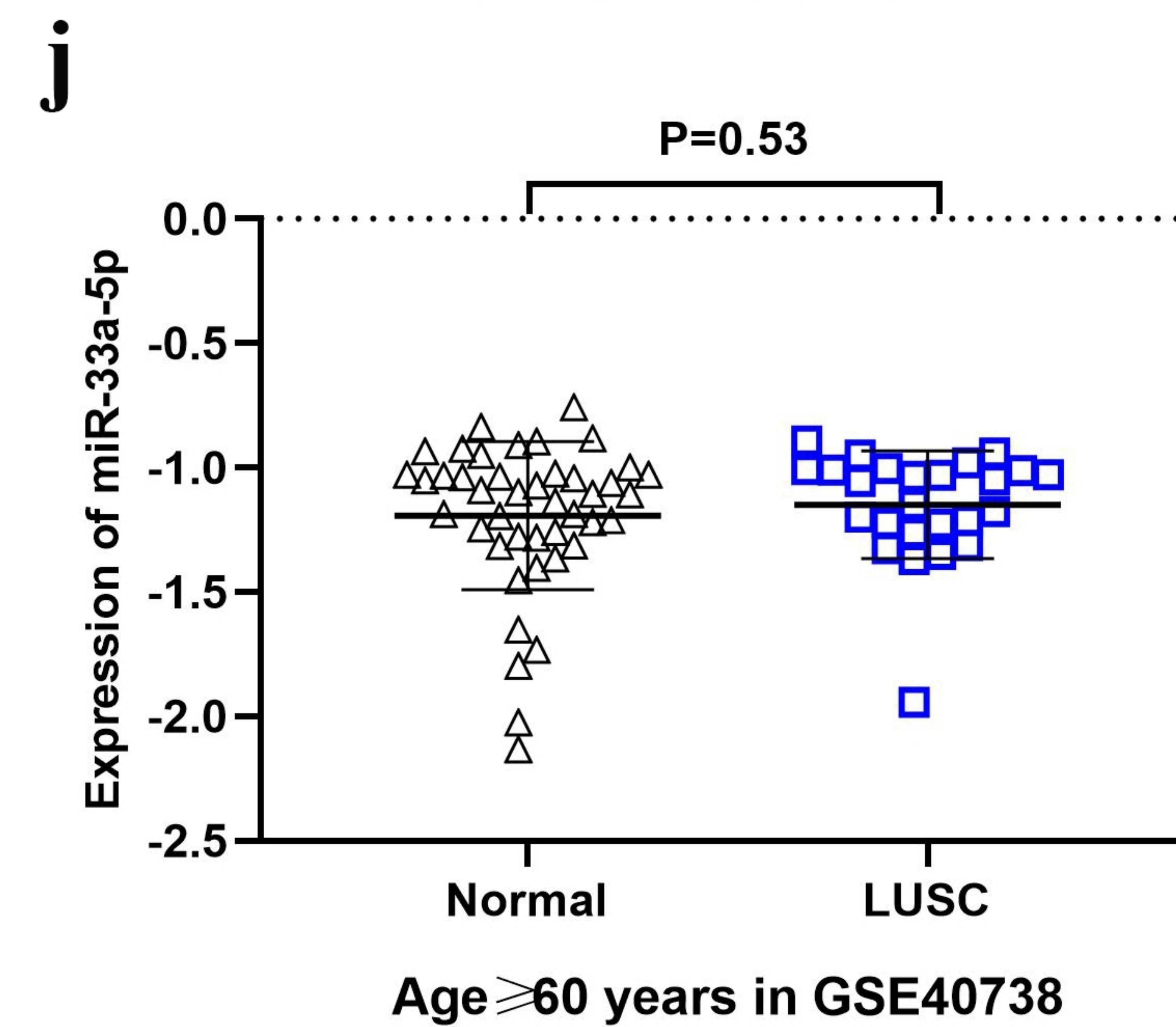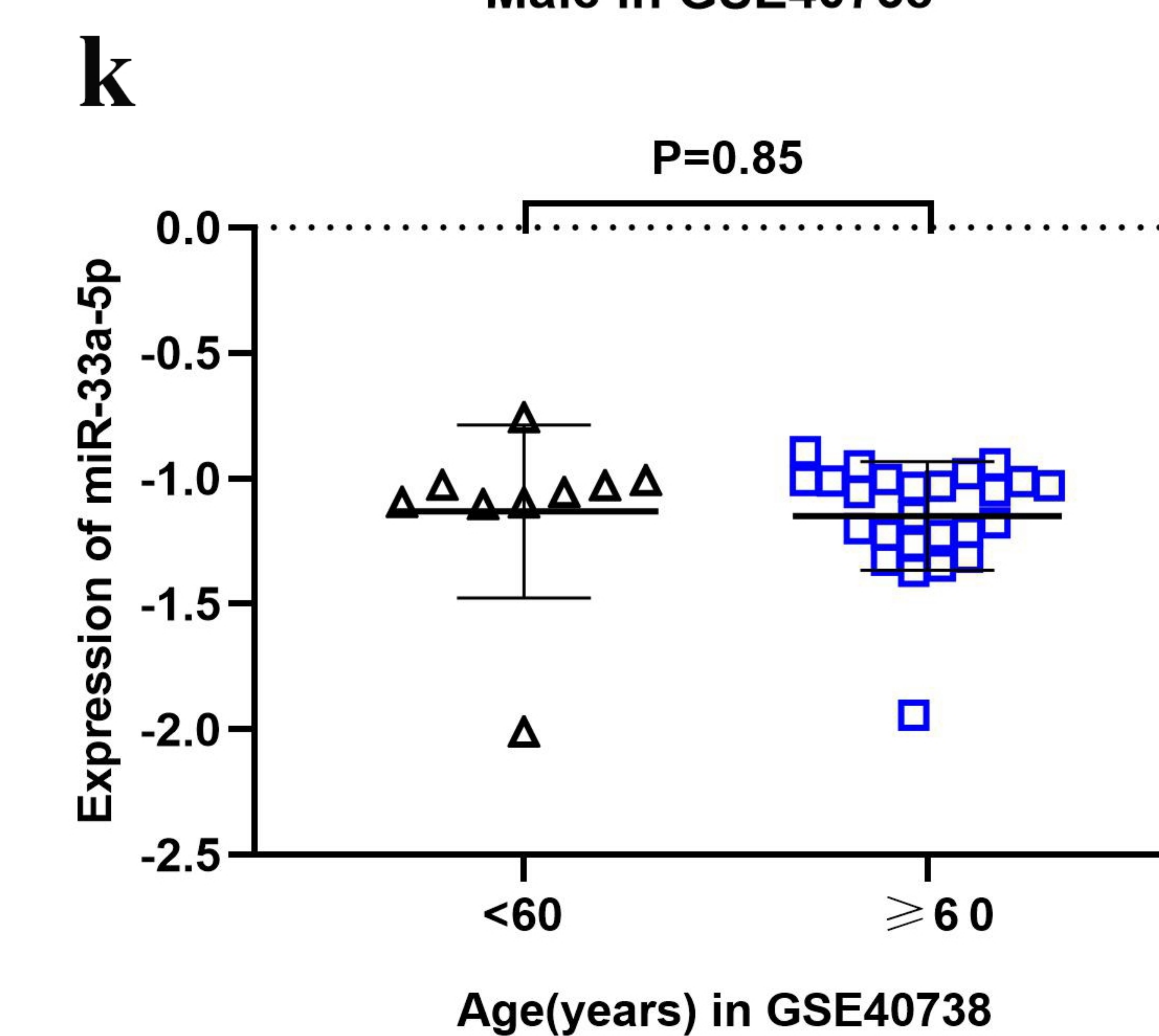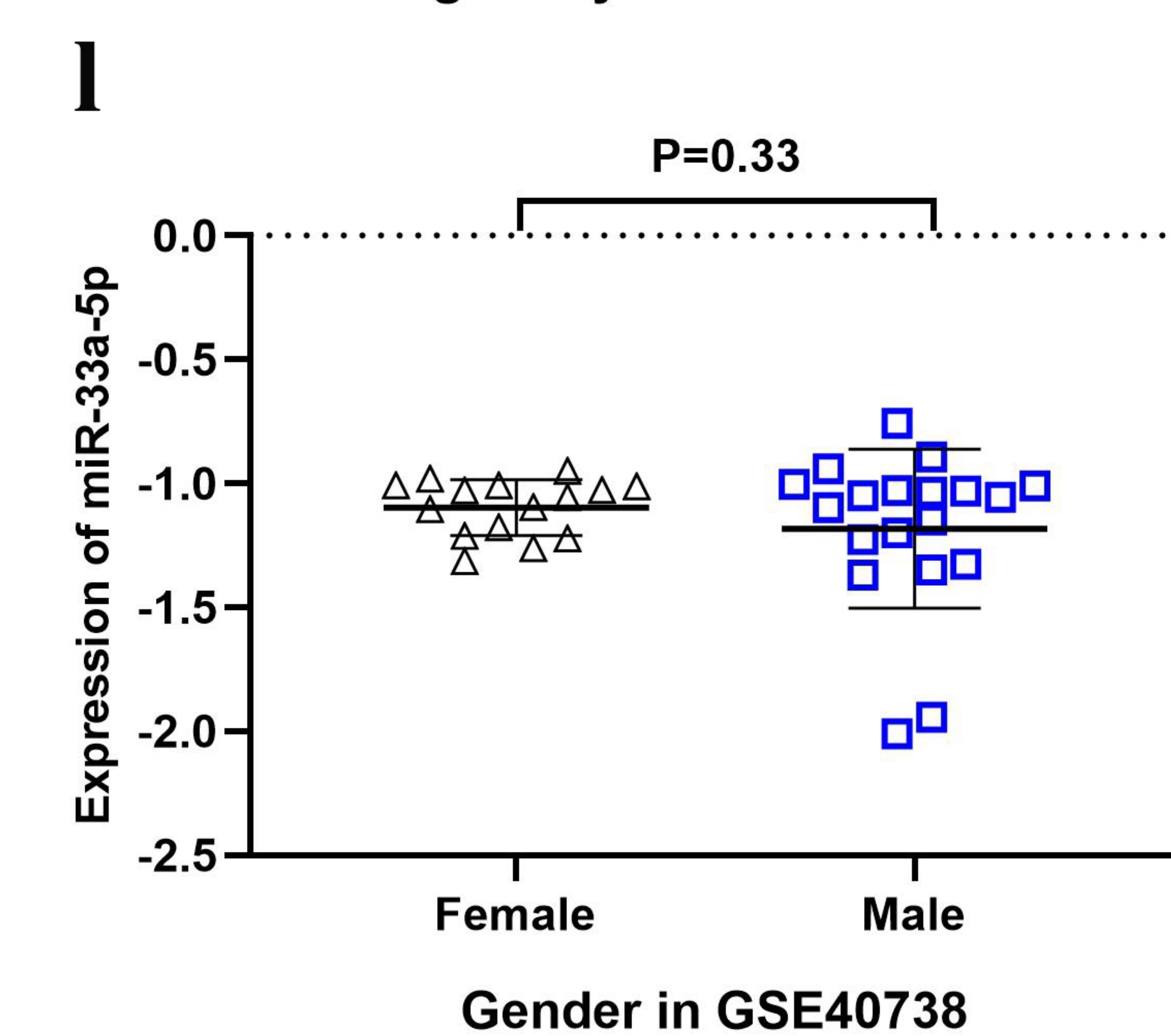

Supplement: Supplementary 1 — Supplementary Figure 1: miRNA-33a-5p expression in different groups of age and gender based on GSE16025 and GSE40738 datasets. (a) The expression of miRNA-33a-5p in LUSC and nontumor tissues for female patients based on GSE16025. (b) The expression of miRNA-33a-5p in LUSC and nontumor tissues for male patients based on GSE16025. (c) The expression of miRNA-33a-5p in LUSC and nontumor tissues for patients with age < 60 based on GSE16025. (d) The expression of miRNA-33a-5p in LUSC and nontumor tissues for patients with age ≥ 60 based on GSE16025. (e) The expression of miRNA-33a-5p for the age of LUSC patients based on GSE16025. (f) The expression of miRNA-33a-5p for the gender of LUSC patients based on GSE16025. (g) The expression of miRNA-33a-5p in LUSC and nontumor tissues for female patients based on GSE40738. (h) The expression of miRNA-33a-5p in LUSC and nontumor tissues for male patients based on GSE40738. (i) The expression of miRNA-33a-5p in LUSC and nontumor tissues for patients with age < 60 based on GSE40738. (j) The expression of miRNA-33a-5p in LUSC and nontumor tissues for patients with age ≥ 60 based on GSE40738. (k) The expression of miRNA-33a-5p for the age of LUSC patients based on GSE40738. (l) The expression of miRNA-33a-5p for the gender of LUSC patients based on GSE40738. Data are expressed as the means ± SD, and P < 0.05 indicates a statistically significant difference when compared to the control. LUSC: lung squamous cell carcinoma. [file 6614331.f1.pdf]

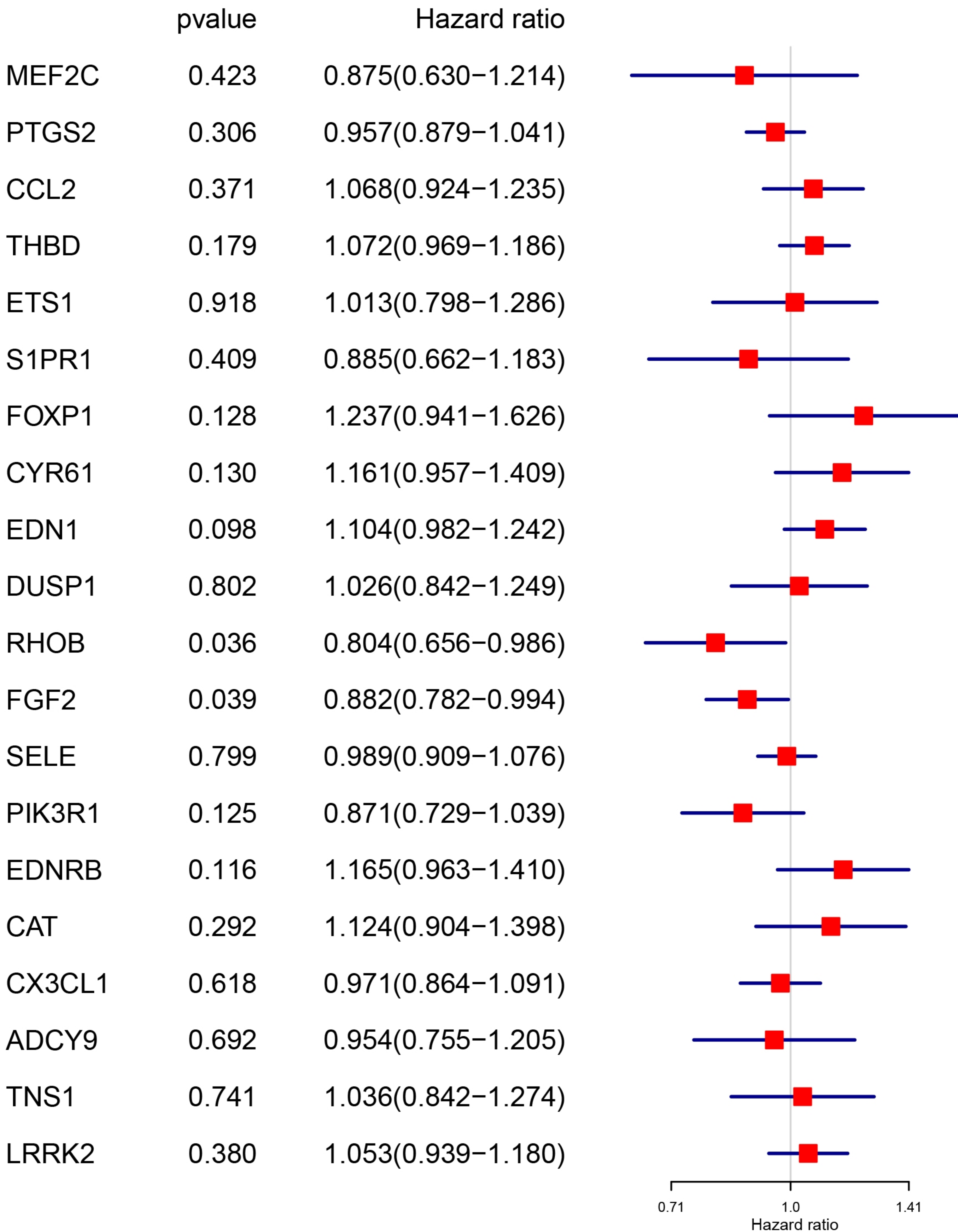

Supplement: Supplementary 2 — Supplementary Figure 2: Cox multivariate regression analysis for the prognostic value of 7 prognosis-related genes and LUSC clinicopathological parameters according to TCGA. P < 0.05 indicates a statistical significance. [file 6614331.f2.pdf]

**a**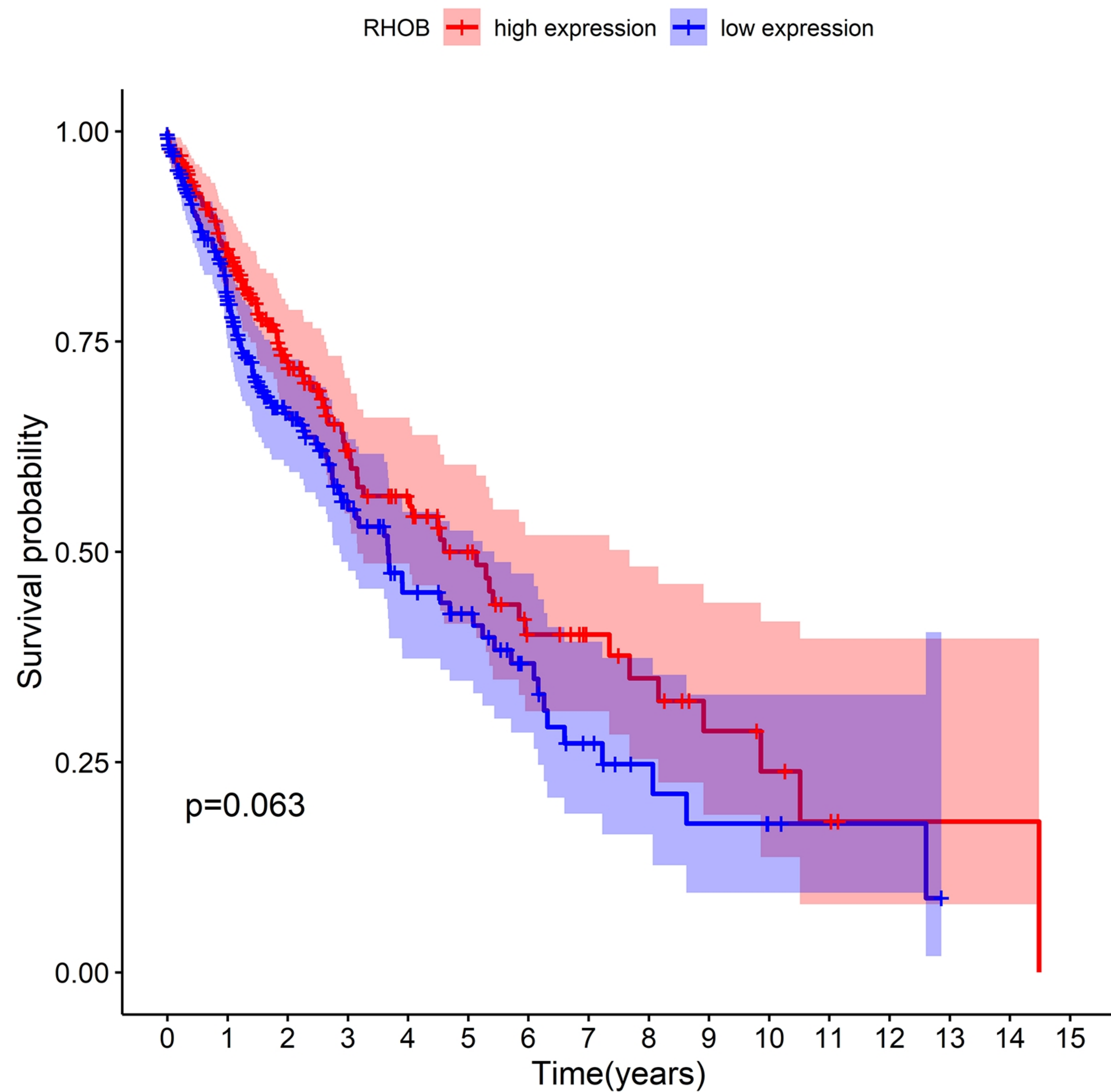**b**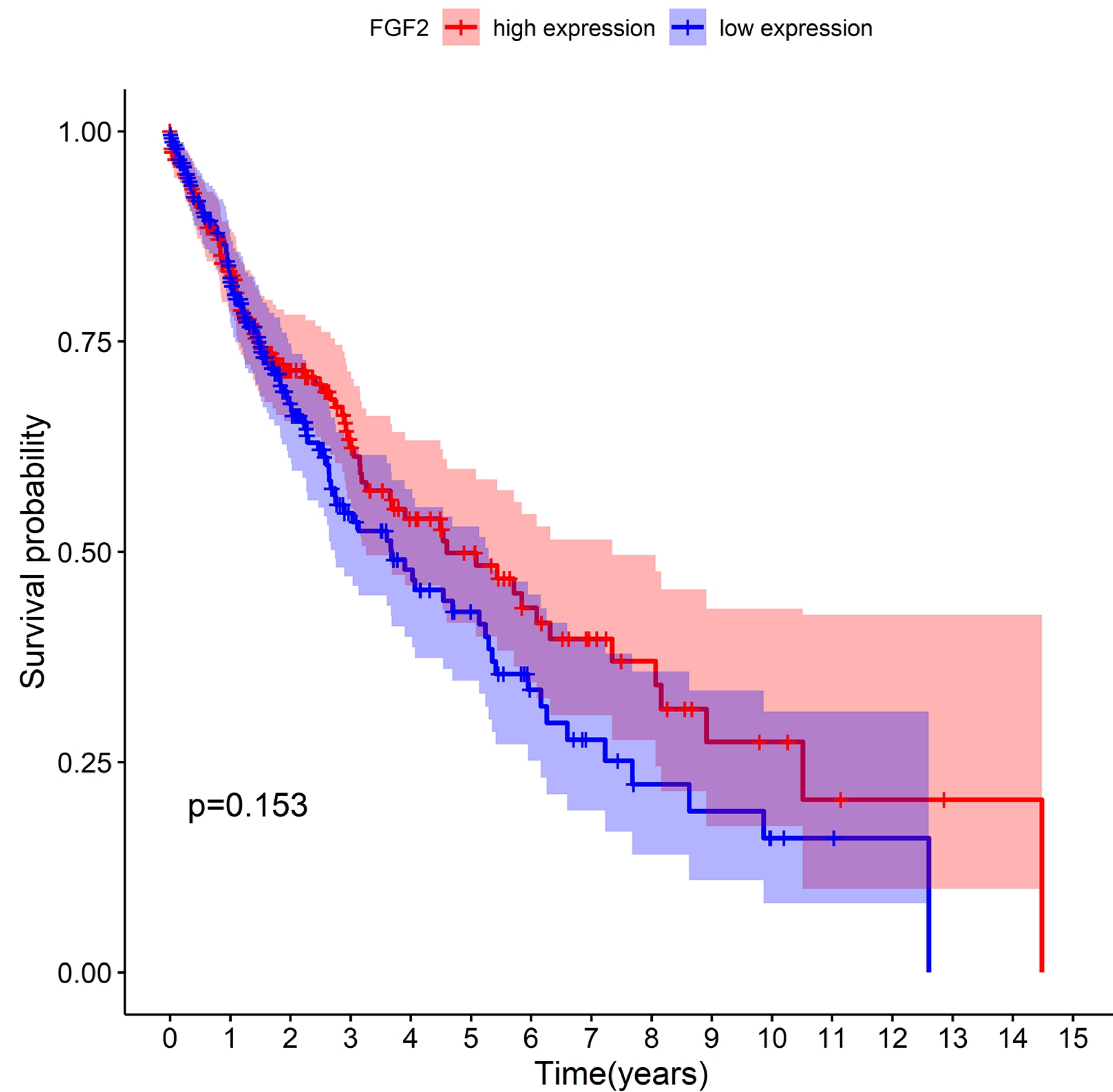

Supplement: Supplementary 3 — Supplementary Figure 3: survival analysis of two prognosis-related genes of miRNA-33a-5p based on TCGA dataset. (a) RHOB. (b) FGF2. [file 6614331.f3.pdf]
